# A new family Lepidocharontidae with description of *Lepidocharon* gen. n., from the Great Barrier Reef, Australia, and redefinition of the Microparasellidae (Isopoda, Asellota)

**DOI:** 10.3897/zookeys.594.7539

**Published:** 2016-05-30

**Authors:** Diana M. P. Galassi, Niel L. Bruce, Barbara Fiasca, Marie-José Dole-Olivier

**Affiliations:** 1 University of L’Aquila, Department of Life, Health and Environmental Sciences, Via Vetoio, Coppito, 67100 L’Aquila, Italy; 2 Museum of Tropical Queensland, Queensland Museum, 70–102 Flinders Street, Townsville, 4810 Australia; Unit for Environmental Sciences and Management and Water Research Group (Ecology), North West University, Potchefstroom 2520, South Africa; 3 Université de Lyon, CNRS, UMR 5023 - LEHNA, Laboratoire d’Ecologie des Hydrosystèmes Naturels et Anthropisés, 6 rue Raphael Dubois, 69622 Villeurbanne, France

**Keywords:** Isopoda, Microparasellidae, Lepidocharontidae new family, Lepidocharon new genus, coral reef, Australia

## Abstract

Lepidocharontidae Galassi & Bruce, **fam. n.** is erected, containing *Lepidocharon* Galassi & Bruce, **gen. n.** and two genera transferred from the family Microparasellidae Karaman, 1934: *Microcharon* Karaman, 1934 and *Janinella* Albuquerque, Boulanouar & Coineau, 2014. The genus *Angeliera* Chappuis & Delamare Deboutteville, 1952 is placed as genus *incertae sedis* in this family. The Lepidocharontidae is characterised by having rectangular or trapezoidal somites in dorsal view, a single free pleonite, a tendency to reduction of the coxal plates, and the unique uropodal morphology of a large and long uropodal protopod on which the slender uropodal exopod articulates separately and anteriorly to the endopod. *Lepidocharon* Galassi & Bruce, **gen. n.** has a 6-segmented antennula, a well-developed antennal scale (rudimentary exopod), long and slender pereiopods 1–7 directed outwards, coxal plates rudimentary, incorporated to the lateral side of the sternites, not discernible in dorsal view, the single pleonite narrower than pereionite 7, scale-like elements bordering the proximal part of male pleopod 1 on posterior side, and stylet-guiding grooves of male pleopod 1 which run parallel to the outer lateral margins of the same pleopod. *Lepidocharon priapus* Galassi & Bruce, **sp. n.**, type species for the genus, and *Lepidocharon lizardensis* Galassi & Bruce, **sp. n.** are described from Lizard Island, northern Great Barrier Reef. The most similar genus is *Microcharon*, both genera sharing the same general organization of the male pleopods 1 and 2, topology and architecture of the stylet-guiding groove of male pleopod 1, morphology of female operculum, presence of 2 robust claws of different lengths on pereiopodal dactylus 1–7, not sexually dimorphic. *Lepidocharon* **gen. n.** differs from *Microcharon* in the shape of the pereionites, very reduced coxal plates, the presence of imbricate scale-like elements bordering the proximal postero-lateral margins of the male pleopod 1, and the topology of the pereiopods, which are ventro-laterally inserted and directed outwards in *Lepidocharon* **gen. n.** and dorso-laterally inserted and directed ventrally in *Microcharon*. *Lepidocharon* shares with the genus *Janinella* the morphology of the tergites and the reduced *lacinia mobilis* of the left mandible, but differs significantly from *Janinella* in having a well-developed antennal scale, very reduced coxal plates also in females bearing oostegites, the general morphology and spatial arrangement of the stylet-guiding groove of male pleopod 1 and the possession of a 6-segmented antennula. The family Microparasellidae is redefined as monotypic, the only genus being *Microparasellus* Karaman, 1933.

## Introduction

Kensley (2001) stated that 84% of the species are endemic to the Indian Ocean overall, but only 18% of the genera. This level of endemicity is generally true for free-living marine Isopoda, and consistent with that observed in the Great Barrier Reef isopod fauna. Data for coral-reef Asellota are few and at present all Great Barrier Reef Asellota are endemic at species level and genus level with the only apparent endemic genus, *Prethura* Kensley, 1982 having been recently reported from Japan (Shimomura and Naruse 2015). Species-level endemicity is high among marine isopods, but generic endemicity is generally low, so the discovery that the two new species from Great Barrier Reef belong to a new genus is noteworthy.

The marine isopod fauna of Queensland is diverse with 343 recorded species. Of that total only 22 species (6.4%) are Asellota, the suborder clearly being under-documented for the region as worldwide (Poore and Bruce 2012, Schotte et al. 2013). They constitute approximately 33% of all isopod species. Of those 22 species, 16 have been described since 2009 (Bruce 2009, Shimomura and Bruce 2012, Bruce and Buxton 2013, Bruce and Cumming 2015) highlighting the potentially high diversity of the suborder (Roberts et al. 2002). Tropical regions from northern and western Australia have an even lower level of recorded species. Given the low level of reporting from the region it is unsurprising therefore to discover both new genera and also families not previously recorded from tropical Australia. The discovery of what appeared to be the first marine Microparasellidae Karaman, 1934 from the Great Barrier Reef (GBR) and Australia was in itself not surprising and furthermore the family was known from coral reefs in New Caledonia (Coineau 1970) and the Caribbean (Kensley 1984).

Describing what proved to be a new genus of Asellota led to a reappraisal of the Microparasellidae, and to the conclusion that the family should be split, with the new genus being placed in the Lepidocharontidae Galassi & Bruce, fam. n. A further three species of Lepidocharontidae have been collected from the Great Barrier Reef, including Heron Island in the south, these being undescribed species of *Microcharon* and *Lepidocharon*, respectively, and a distinctive new species of uncertain generic identity, yielding a total of at least five species in three genera for the GBR. The presence of this number of species suggests that with appropriate collecting in suitable habitats this family may be more diverse in marine habitats that previously believed.

## Methods

Collection methods have recently been described by Bruce (2015) and Bruce and Buxton (2013). CReefs samples were all preserved in high-grade ethanol (a requirement of the CReefs program), without first fixing in formalin. Drawings and measurements were made using a camera lucida on a Leica DM 2500 phase contrast and interferential microscope. Some details gained from scanning electron microscopy (SEM) are added to line drawings. Scanning Electron Microscope specimens of *Lepidocharon priapus* sp. n. were dehydrated in a graded ethanol series, critical point dried in a Balzers Union CPD 020 apparatus and sputter coated with gold in a Balzers Union SCD 040. Observations were made with a Philips SEM XL30 CP scanning electron microscope.

Sampling was carried out under GBRMPA Permit G08-27858.1 and General Fisheries Permit (QLD DPI) 95152.

The type material is deposited in the Museum of Tropical Queensland, Australia (MTQ).

## Results

### Taxonomy
Asellota Latreille, 1803
Janiroidea Sars, 1897

#### Lepidocharontidae

Taxon classificationAnimaliaIsopodaLepidocharontidae

Galassi & Bruce fam. n.

http://zoobank.org/B21B701E-73AE-4433-84C3-1EA34618814B

##### Diagnosis.

**Male.** Body dorsally flat, slender, ~4–10× long as wide, without chromatophores; somites all subsimilar in width, somites sub-rectangular or trapezoidal, lateral margins of head and pereionites sub-parallel. Pleon of one segment, with free lateral margins. Head with weak or absent rostrum, without pseudorostrum. Eyes absent. Antennula with maximally 4 flagellar articles. Antenna flagellum longer than podomeres. Antennal scale (rudimentary exopod) present, even if more or less developed among genera. Mandible incisor with 2 to 8 cusps; molar process subconical, without grinding surface, with apical unequal smooth and pinnate setae; spine row and *lacinia mobilis* present, the latter only on left mandible. Maxilliped slender, covering entire mouthpart field, endite distal margin narrowly rounded; epipod slender, quadrate or distally acute; palp composed of 5 articles; 2 stiff pectinate setae always present on maxilliped distal article. Pereiopods 1–7 subsimilar, always without subchela; all pereiopods with 2 dactylar claws; pereiopods articulating dorso-laterally or laterally, and projecting ventrally (in *Janinella* and *Microcharon*) or outwards (in *Lepidocharon*). Penial processes with openings coalescent and medial. Male pleopods 1 and 2 not operculate; male pleopod 1 distally rounded or subtruncate, with or without acute distolateral lobes; proximal part of the pleopod with or without scale-like elements on postero-lateral margins; stylet-guiding grooves running parallel to the lateral free distal margin of pleopod 1 and folded by a hyaline lamella (transversal and unfolded in *Janinella*); pleopod 3 endopod with 3 plumose setae (marine taxa) or without plumose setae (freshwater taxa), exopod slender. Pleopod 4 globular, pleopod 5 absent. Uropods biramous, ventrally inserted on pleotelson, protopod large, *c.* 0.5–1.3 as long as pleotelson; protopod length/width ratio *c.* 2.5–4.5; rami slender with exopod articulating anteriorly to endopod. Anus terminal, not covered by pleopods. Anus outside pleopodal chamber, between bases of uropodal protopods.

**Female.** Operculum (pleopod 2) from sub-quadrate (as long as wide) to more than 2 times longer than wide, with free distal margin deeply incised medially, faintly incised, or without medial incision, armed with 4 or 2 setae, or unarmed.

##### Genera included.

*Microcharon* Karaman, 1934; *Janinella* Albuquerque, Boulanouar & Coineau, 2014; *Lepidocharon* Galassi & Bruce, gen. n.

##### Genus *incertae sedis*.

*Angeliera* Chappuis & Delamare Deboutteville, 1952.

##### Remarks.

Wilson and Wägele (1994) and Wilson (1994) critically discussed the status of the Microparasellidae in relation to the Janiridae on a cladistic phylogenetic basis. Wilson and Wägele (1994) in their review of the Janiridae analysed the status of the family Microparasellidae in detail, reviewing the history of the debate over the status of the family. Wilson (1994) also included the Microparasellidae in his cladistic analysis of the phylogeny of the Janiridae. Wilson and Wägele (1994: page 721) stated “*The family concept of the Microparasellidae may be open to challenge because* Microparasellus *is distinct from the other three genera in these autapomorphies*”, these being the differences in the somatic and uropodal morphology. These authors went on to say “*the composition of this family will require further study*.” In Wilson’s (1994) analysis, there were no supporting apomorphies for the Microparasellidae as then constituted, but there were separate supporting apomorphies for the genus *Microparasellus* and the clade holding the remaining genera, strongly suggesting that potentially these were two monophyletic clades, albeit the Microparasellidae being monogeneric. The principle basis for this is that each group had unique and derived uropod morphology and substantial differences in body morphology.

The description of the new genus *Lepidocharon* Galassi & Bruce, gen. n. led to a re-appraisal of the taxonomic status of the Microparasellidae and its constituent genera. We conclude that the Microparasellidae is a mono-generic family supported by a prominent acute or narrowly rounded rostrum, the antennal flagellum shorter than podomeres, all somites with straight lateral margins that also have scales, an indisputable ventral position of the pereiopods, the unique uniramous and short uropods (see Appendix 1). The remaining genera are housed in the new family Lepidocharontidae fam. n., the diagnostic characters being the elongate body (up to 10× as long as maximum width), a weak or absent rostrum, the antennal flagellum longer than podomeres, the pereionites rectangular or trapezoidal in dorsal view, with sub-parallel lateral margins, a lateral or dorso-lateral position of the pereiopods, a tendency to reduction of pereiopodal coxal plates, and the uropod with a large protopod with the exopod articulating anteriorly and separately to the endopod.

Within the family Lepidocharontidae there is great uniformity of the diagnostic characters among all the genera. The genus *Angeliera* has been placed *incertae sedis* in the Asellota on the basis of marked differences in several morphological features that set this genus far away the basic body plan observed in Lepidocharontidae.

The largest genus in the Lepidocharontidae is *Microcharon* with 77 species, both marine and freshwater. Many species lack full descriptions, and there are inconsistencies in the distribution of certain characters within the genus. A dorsal view of the head is not routinely figured; when figured, it can be seen that some species do have a rostral point or rostrum, while others have the anterior margin of the head weakly concave.

The generic name *Microcharon* is unavailable under the ICZN (1999)’s rules because the genus was established by Karaman (1934) without type species designation. This prevents the use of “Microcharontidae” (from the most speciose and well-known genus *Microcharon*) as the family name because ICZN (1999)’ article 13.2 unambiguously states: “To be available, every new family-group name published after 1930 must satisfy the provisions of Article 13.1 and must be formed from an available genus-group name then used as valid by the author in the family-group taxon [Arts. 11.7.1.1, 29]”. Therefore we here propose the name Lepidocharontidae fam. n.

The family name Microparasellidae was first introduced by Karaman (1934: page 44) when describing the genus *Microcharon*, although the family had been earlier diagnosed by Karaman (1933: page 17) with the accompanying statement “Microparasellus *n. fam., n. gen.*”, but without type-species designation for the genus *Microparasellus* (see Karaman 1933). According to the ICZN (1999: Article 13.2) the family name Microparasellidae proposed by Karaman (1934) is thus a *nomen nudum*, because the family was erected on the unavailable generic name *Microparasellus* Karaman, 1933 that lacked type-species designation. Nevertheless, Article 13.2.1 states that “A family group name first published after 1930 and before 1961 which does not satisfy the provisions of Article 13.1 is available from its original publication only if it was used as valid before 2000, and also was not rejected by an author who, after 1960 and before 2000, expressly applied Article 13 of the then current editions of the Code”. The family name was considered valid until 2000, and for this reason it is an available name as Microparasellidae Karaman, 1934. The family name Microparasellidae is then valid.

Conversely, we provisionally maintain current and common usage of the names *Microparasellus* and *Microcharon*, and this is discussed in more detail together with a new diagnosis for the Microparasellidae (see Appendix 1). As the nomenclature within the family Microparasellidae is well established and widely used, a proposition (Galassi and Bruce in preparation) will be submitted to the ICZN Commission for maintaining the stability of the current nomenclature and related authorities.

##### Key to Microparasellidae and genera of Lepidocharontidae fam. n.

**Table:** 

1	Uropods uniramous, short; antennal podomeres longer than flagellum, scale missing; head with prominent rostrum	**Microparasellidae (*Microparasellus*)**
–	Uropods biramous, large; antennal podomeres shorter than flagellum; rostrum small or absent	**2**
2	Pereionites 1–7 cylindrical, free pleonite as wide as pereionite 7	***Microcharon***
–	Pereionites 1–7 dorsally flat and trapezoidal, except pereionite 4; free pleonite narrower than pereionite 7	**4**
3	Male pleopod 1 with transverse stylet-guiding grooves, unfolded; proximal postero-lateral margins of male pleopod 1 without scale-like elements	***Janinella***
–	Male pleopod 1 with distal stylet-guiding grooves parallel to the lateral margins, folded by hyaline lamella posteriorly; proximal lateral margins of male pleopod 1 armed with scale-like elements	***Lepidocharon* gen. n.**

#### Lepidocharon

Taxon classificationAnimaliaIsopodaLepidocharontidae

Galassi & Bruce gen. n.

http://zoobank.org/6263F663-8F45-43A2-8AFC-678DE31BD450

##### Type species.

*Lepidocharon priapus* Galassi & Bruce, sp. n.; here designated.

##### Other species.

*Lepidocharon lizardensis* Galassi & Bruce, sp. n.

##### Diagnosis.

**Male.** Body slender, 8.5–9.7 as long as wide. Free pleonite narrower than pereionites and pleotelson, visible in dorsal view. Cephalon medio-frontal margin not produced, anterior margin straight, rostrum absent. Pereionites 1–3 anteriorly widest, with distinct anterolateral angle, pereionite 4 sub-rectangular, pereionites 5–7 posteriorly widest, lateral margin with distinct posterolateral angle. Pereionites dorsally ornamented by paired setae. Cuticle with small semicircular thickening present or absent both dorsally and ventrally. Antennula 6-segmented; long aesthetascs on articles 5 and 6, long brush seta on article 2 extending to tip of article 6. Antenna with 6 podomeres; article 3 with long blade-like or candle flame-like scale, reaching article 5; lateral margin with 2 setae; flagellum with 8–12 articles.

Mandible palp article 1 unarmed, article 2 with 2 stiff spinulose setae, article 3 with 5 stiff spinulose setae. Right mandible: incisor with 6 to 9 cusps; *lacinia mobilis* absent; molar process conical, with 3 setae. Left mandible: incisor with 2 to 3 cusps; *lacinia mobilis* present and produced in 2 cusps; molar process conical, with 3 setae. Maxillula: mesial lobe slender and tapering at distal part, bearing 1 short apical seta accompanied by subapical shorter setae and lateral thin and short setae. Lateral lobe sub-rectangular in shape. Apical setation composed by a variable number of setae. Maxilla: mesial ramus with 8–9 setae; 1 apical comb-like seta, strong, unipinnate and ornamented with fine regularly-spaced setules parallel to one another. Lateral rami close-set, each bearing 4 slender and simple setae, respectively. Maxilliped palp wider than endite, mesial margin of articles 2 and 3 expanded. Pereiopods all subequal in length, all subsimilar in size and general morphology; all with 2 dactylar claws; pereiopod 1 dactylar claws subequal; pereiopods 2–7 superior claw slender (3.8–4.3× basal width), inferior claw robust (2.3–2.6× basal width). Coxae rudimentary reduced to small sclerites, not discernible in dorsal view, coalescent to body wall of the sternites, located on the anterior margin of the concavity which houses the propodus, and apparently not articulated to the sternites.

Pleotelson 1.3–1.7 as long as wide, 1.7–1.9 as long as pereionite 7. Penial papillae opening on postero-medial margin of sternite 7. Pleopod 1 rami proximally fused; proximolateral margins with cuticular imbricate scales on posterior side; distolateral margins convex; stylet-guiding groove represented by a folded hyaline lamella running sub-parallel to free lateral margin of rami; pleopod 1 transverse stylet-guiding grooves absent, unlike *Janinella*. Pleopod 2 stylet long and slender, of variable length. Pleopod 3 endopod bearing 3 distal plumose setae; exopod elongate, lateral margin with thin setae, article 2 with 1 subapical seta; pleopod 4 rudimentary, ovoid, uniramous. Uropodal protopod as long as the pleotelson, slender (length/width ratio: 4.3), not sexually dimorphic, with long and slender exopod and endopod.

**Female.** As for the male, except for sexual characters. Operculum (pleopod 2) longer than broad, with surface smooth or with semicircular thickening, with distal margin faintly incised, and 4 apical setae.

##### Etymology.

The generic name is derived from the ancient Greek name *λεπις*, *λεπιδος* meaning “scale”, which refers to the unique rim of scale-like elements bordering the proximal part of the first male pleopod on the posterior side, combined with the mythological name *Charon*, *Charontis* referring to the Ferryman of Hades. Gender: masculine.

##### Remarks.

*Lepidocharon* gen. n. is most similar to the genus *Microcharon*, the two genera sharing the following characters: well-developed uropods with slender endopod and exopod; pereiopodal coxal plates not discernible in dorsal view, small, incorporated to the sternite body wall; male pleopod 1 with similar general organization, the distal lateral lobe with a folded hyaline lamella (stylet-guiding groove) running almost parallel to the lateral margins of the pleopod (this orientation and structure of the stylet-guiding groove appears different from that of *Janinella*, where a transversal and oblique groove hosts the stylet of the male pleopod 2 which seems not to be enveloped by a hyaline lamella); pleopod 2 identical in the development of both exopod and endopod, the latter ending with a stylet of different length depending on the species; penial papillae small and located at the posteromesial margin of sternite 7; female operculum as long as the pleotelson, faintly incised, bearing 4 apical setae (a condition shared by some predominantly marine, and rarely freshwater, *Microcharon* species).

*Lepidocharon* gen. n. shares with *Janinella* the morphology of the tergites, the first three pereionites with antero-lateral protrusions, the fourth sub-rectangular in shape, and the last three postero-laterally protruded, together with the lateral insertion of pereiopods 1–7 oriented outwards (*vs.* ventrally in *Janinella*, see Albuquerque et al. 2014); pereiopodal coxal plates small in *Janinella*, very reduced and incorporated to body wall in *Lepidocharon* gen. n. The female operculum of *Lepidocharon* is more than twice as long as wide, as long as the pleotelson, only faintly incised and bearing 4 apical setae, the mesial pair being close-set. These apical setae resemble that of *Janinella* species, where only the two close-set setae are present.

*Lepidocharon* gen. n. differs from all the other lepidocharontid genera by the combination of the following morphological characters: (1) the unique presence of scale-like elements bordering the postero-lateral margins of the proximal part of the male pleopod 1; (2) pereionites with coxal plates hardly discernible, small, and incorporated into the sternite body wall, not visible in dorsal and lateral views; (3) the long and slender pereiopods that are inserted laterally and directed outwards; (4) pereionites that are not cylindrical, except pereionite 4 (*vs.* cylindrical in *Microcharon*); (5) the presence of elongate antennal scale (*vs.* rudimentary in *Janinella*, and generally reduced in *Microcharon*); (6) the mandible incisor with up to 9 cusps (lower number of cusps in *Microcharon* and *Janinella*).

*Microcharon galapagoensis* Coineau & Schmidt, 1979 is closer to *Lepidocharon* gen. n., especially in the general body morphology, pereionite shape, slender pereiopods directed outwards, a long antennal scale, and slender and elongate uropods; however the unique scale-like elements of the male pleopod 1 of *Lepidocharon* are not present in this species. These scales are not easily detected under optical microscopy (if not at 100× magnification), nor were they seen using SEM because located on the posterior side of the pleopod.

The mid-section of the male pleopod 1 shows lateral margins expanded in both species of *Lepidocharon* gen. n. In contrast to the new genus, *Microcharon galapagoensis* shows a 5-segmented antennula. Coineau and Schmidt (1979) had informally proposed the creation of an intermediate group between the genus *Microcharon* and *Janinella* (formerly *Paracharon* Coineau, 1970) for *Microcharon galapagoensis* Coineau & Schmidt, 1979 (from marine interstitial habitats in the Galapagos), *Microcharon salvati* Coineau, 1970 (from coral sands in New Caledonia) and *Microcharon herrerai* Stock, 1977 (from brackish groundwater in the Netherlands Antilles) (Stock 1977).

#### Lepidocharon priapus

Taxon classificationAnimaliaIsopodaLepidocharontidae

Galassi & Bruce sp. n.

http://zoobank.org/C6CDCAD6-3118-4E4A-A918-9B922E58E17D

Figures 1 , 2 , 3 , 4 , 5 , 6 , 7 , 8 , 15A

##### Material examined.

*Holotype* here designated. Adult ♂ (1.3 mm) completely dissected and mounted in polyvinyl lactophenol on one slide (MTQ W28329), 17 February 2009, coll. N.L. Bruce and M. Błażewicz-Paszkowycz.

Type-locality: Australia, Great Barrier Reef, off Coconut Beach, Lizard Island, reef front, sand adjacent to bommies, 4 m, stn LIZ 09–09A, 14.68441°S, 145.47197°E.

*Paratypes.* 2 ♂♂ (0.9, 1.1 mm), 2 ♀♀ (1.1, 1.4 mm), Great Barrier Reef, Australia, completely dissected and mounted in polyvinyl lactophenol; 2 ♂♂, 1 ♀, same data as holotype preserved in alcohol; 1 ♂, 1 ♀ mounted on SEM stubs (all MTQ W30933).

##### Etymology.

The epithet is derived from the god *Priapus* (Πρίαπος) of the Greek mythology. He was considered the protector of livestock, fruit plants, gardens and male genitalia. He was famous for his largely phallic character and the specific names is here referring to the extraordinary length of the stylet of male pleopod 2.

##### Description of male.

**Figure 1.**
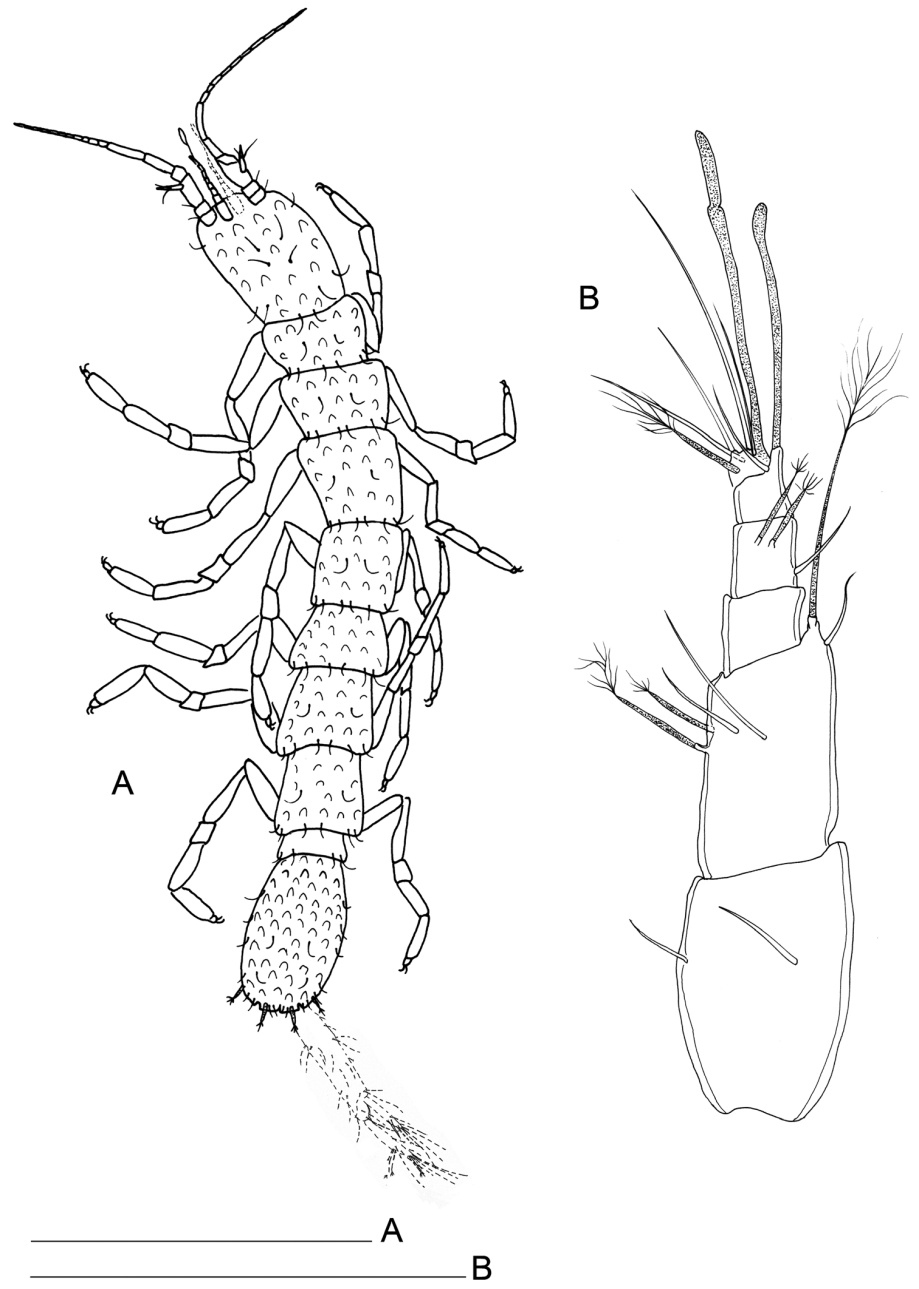
*Lepidocharon priapus* gen. n., sp. n. ♂ holotype. **A** habitus **B** antennula (scale bars: **A** 0.5 mm; **B** 0.1 mm).

**Figure 2.**
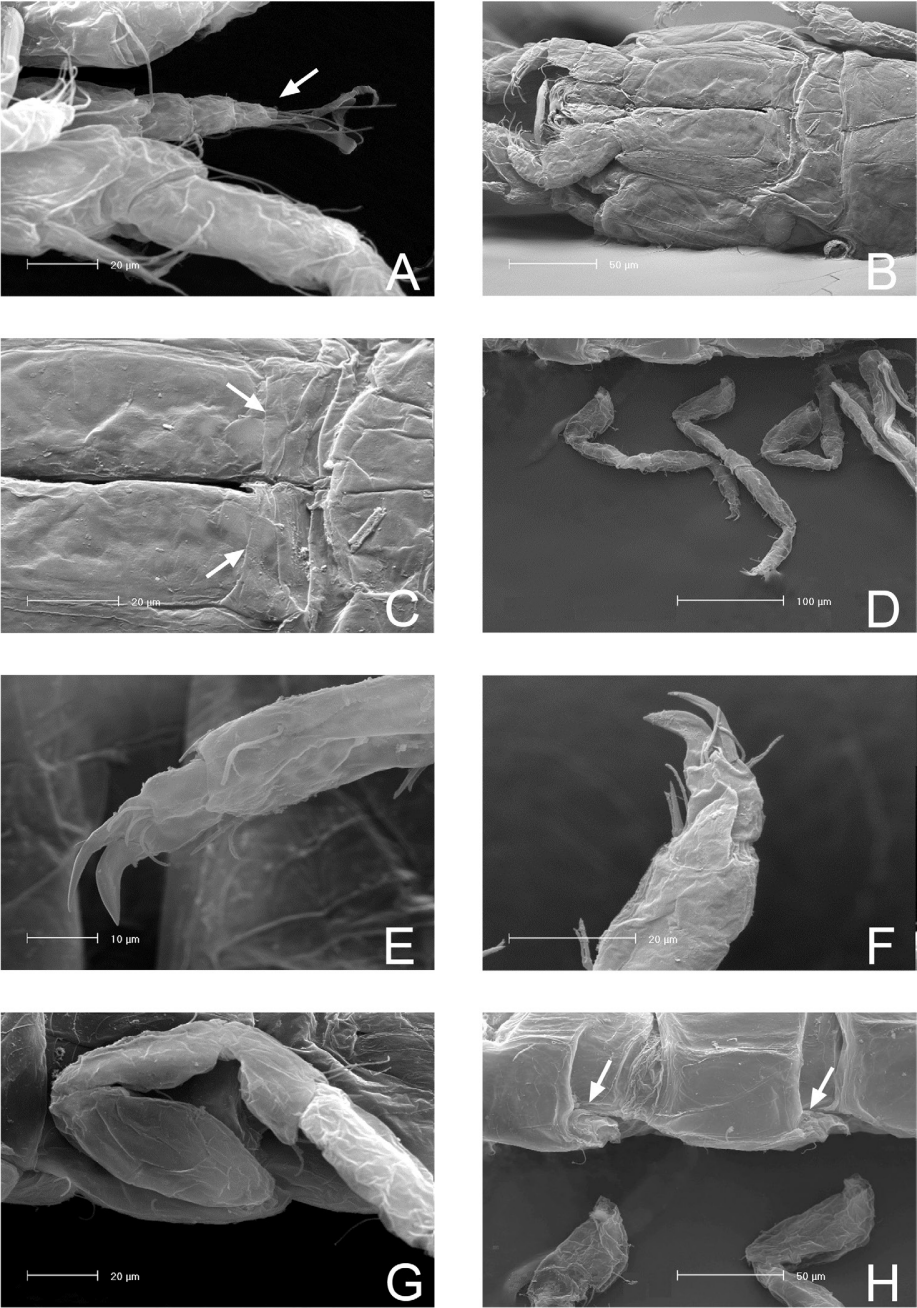
SEM micrographs of *Lepidocharon priapus* gen. n., sp. n. ♂ paratype. **A** antennula, sixth segment arrowed **B** maxillipeds, general view **C** maxilliped endite and rudimentary sympod (?) arrowed **D** coxal plates of pereionites 5–7, ventral view **E** pereiopod 1, detail of dactylus and reduced sclerite **F** pereiopod 7, detail of dactylus and articulated sclerite **G** pereiopods, lateral view **H** rudimentary coxal plates, ventral view, arrowed.

Body length, measured from tip of cephalon to end of pleotelson, from 0.9 to 1.3 mm (n=6). Body 7.0–8.0 times longer than wide, dorsally flat. Cephalon (Fig. 1A) longer than wide (length/width ratio: ~1.3), as large as pereionites, lateral margins sub-parallel. Pereionites 1–3 with anterolateral margins of tergites protruding; pereionite 4 rectangular, without tergite protrusions, pereionites 5–7 with posterolateral margins of tergites protruded. Pereiopods inserted close to lateral closure of tergites (Figs 1A, 2G–H) and extended outwards, coxal plates not discernible on dorsal and lateral views, small, totally incorporated to the border of the concavity housing the pereiopods (Fig. 2D, G–H).

**Figure 3.**
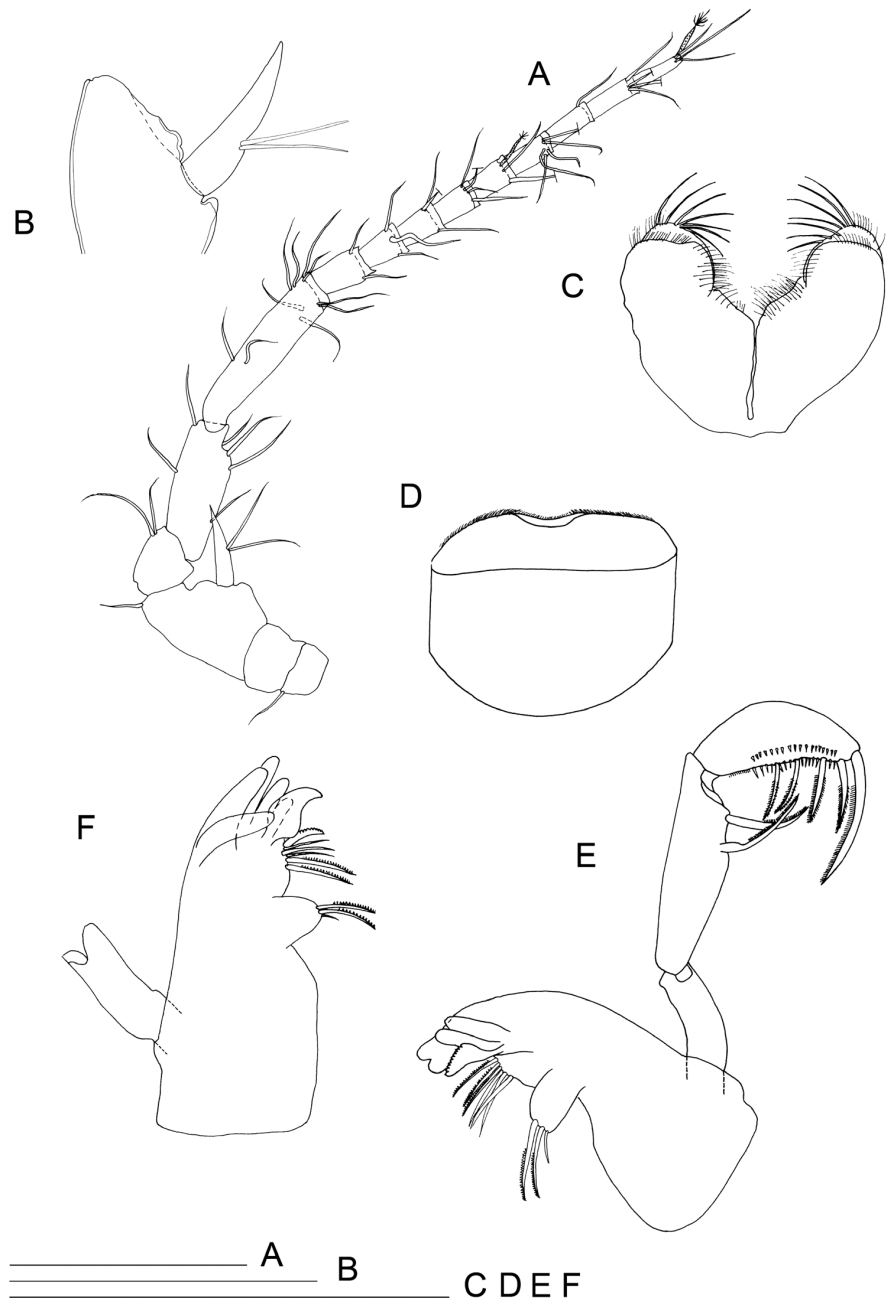
*Lepidocharon priapus* gen. n., sp. n. ♂ holotype. **A** antenna **B** detail of the antennal scale **C** paragnaths **D** labrum **E** left mandible and maxilliped **F** right mandible (scale bars: 0.1 mm).

Paragnaths (Fig. 3C) large, free distal margin with long slender simple setae and marginal and submarginal rows of scale setae. Labrum ovoid (Fig. 3D), ornamented with fine spines on free anterior margin.

Antennula (Fig. 1B) composed of 6 articles; article 1 1.5 as long as wide, directed anteriorly, with 2 simple setae; article 2 1.5 as long as wide, 0.8 as wide and 0.8 as long as article 1, with 4 setae inserted at distal third of article, 2 of which penicillate setae plus 1 long penicillate seta inserted on a lateral protrusion of article, accompanied by a short and thin naked seta on its basis; article 3 unarmed; article 4 with 1 lateral seta and 2 surface penicillate setae; article 5 shorter than article 4, bearing 1 long aesthetasc and 1 simple seta at basis of aesthetasc-bearing protrusion; article 6 short, 0.5 as long as article 5 and clearly articulated with article 5 (Fig. 2A), bearing 5 setae in total, two surface seta (one of which penicillate), one apical and one subapical simple setae; one long aesthetasc and a robust and long seta apically, both incorporated to article 6.

Antenna (Fig. 3A–B) with 6 podomeres, 2 proximal articles short and stout, article 1 with 1 apical outer seta; article 2 naked; article 3 robust, length/width ratio: 1.25, with mesial apical seta and long exopod quite overreaching segment 4, knife-blade shaped and bearing 2 long and thin setae inserted on lateral margin at middle of exopod; article 4 stout and curved outward, 0.5 as long as article 3, with 2 apical mesial setae; articles 5 and 6 slender and long, article 6 longest, bearing 6 and 10 simple setae, respectively; flagellum composed of 9 articles in holotype; 11 articles in one male paratype; all flagellar articles with distal hyaline lamella partially covering insertion of following article, all armed with setae on distal margin, except article 9 ending with 3 simple and 1 penicillate setae.

Mandible palp (Fig. 3E) on short cuticular projection; palp article 1 without setae, article 2 longest, about 2.5 times longer than wide, with 2 pinnate robust setae laterally, their insertion more or less coalescent with article; article 3 curved laterally, with 5 pinnate robust setae, distalmost seta longest; 2 cuticular comb rows on lateral margin of article. Left mandible (Fig. 3E): incisor with two strong and large cusps; *lacinia mobilis* as in the genus; molar process with two long unipinnate setae accompanied by 1 short simple spine. Between *lacinia mobilis* and molar process 3 thin, long and simple spines and 3 unipinnate spines are present, 1 modified seta, cockscomb-shaped, close to *lacinia mobilis*, with total of 7 elements. Right mandible (Fig. 3F) incisor with 6 robust cusps; 2 long unipinnate and 1 short simple seta. Between incisor and molar process 6 spines are inserted, apicalmost robust, curved and unipinnate, 3 naked and 2 unipinnate spines.

**Figure 4.**
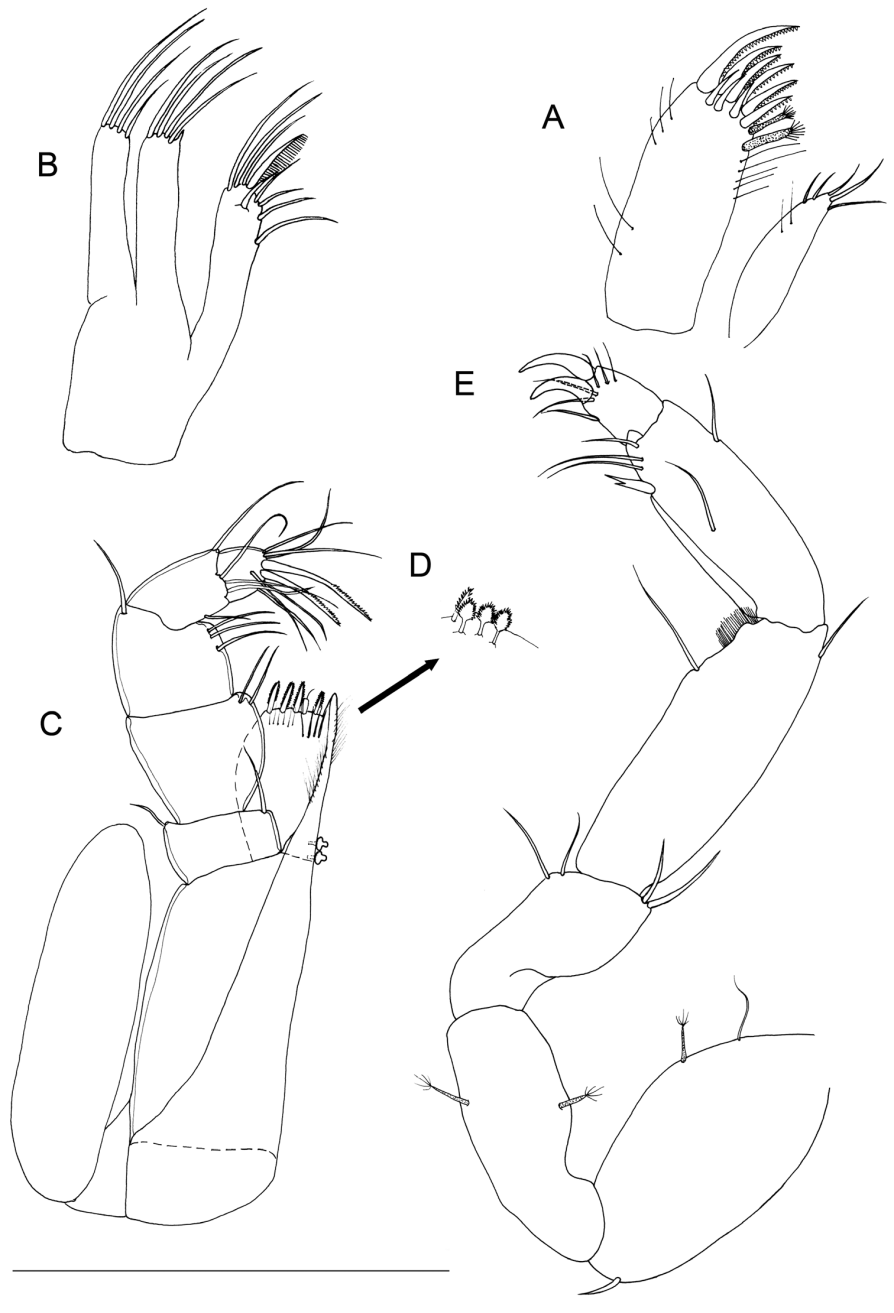
*Lepidocharon priapus* gen. n., sp. n. ♂ holotype. **A** maxillula **B** maxilla **C** maxilliped **D** maxilliped, detail of the subapical setae morphology of the endite **E** pereiopod 1 (scale bar: 0.1 mm).

Maxillula (Fig. 4A): mesial lobe slender and tapering at distal part bearing 1 short apical seta accompanied by 2 subapical shorter setae and two lateral thin and short setae. Lateral lobe sub-rectangular in shape, bearing scale-like elements on both lateral and mesial margins. Apical setation composed by 11 elements; 3 simple setae in subapical position (surface apical setae); 2 mesial setae with apical tuft; remaining 6 distal setae unipinnate.

Maxilla (Fig. 4B): mesial ramus with 9 setae, 3 naked and slender setae on mesial margin, 1 surface seta short; 5 apical setae, mesialmost and 3 lateralmost apical setae simple and slender; second apical setae (starting from mesial margin) comb-like, strong, unipinnate and ornamented with fine regularly-spaced setules parallel to one another. Lateral rami close-set, each bearing 4 slender and simple setae, respectively.

Maxilliped (Figs 4C, D, 2B, C): palp robust and curved inwards; article 1 sub-rectangular in shape, bearing 1 mesial and 1 lateral setae; article 2 longest, robust, rounded on mesial margin, with 2 setae at distomesial angle; article 3 shorter than article 2 with 1 distolateral and 3 mesial setae; article 4 angled mesially, with 1 seta on distolateral margin, 1 seta on mesial margin and 3 setae on distal margin; article 5 about 0.5 as long as article 4, with 8 apical setae, of which 6 are simple slender setae and 2 are stiff pectinate setae. Endite almost reaching end of palp article 2; mesial margin ending in a pointed protrusion, with numerous hair-like setules and 2 coupling hooks medially; apical free distal margin with 4 bipinnate, spine-like, stout setae and 1 simple non-tapered seta; 3 subapical fan setae are present; epipod ovoidal, reaching distal part of palp article 1.

**Figure 5.**
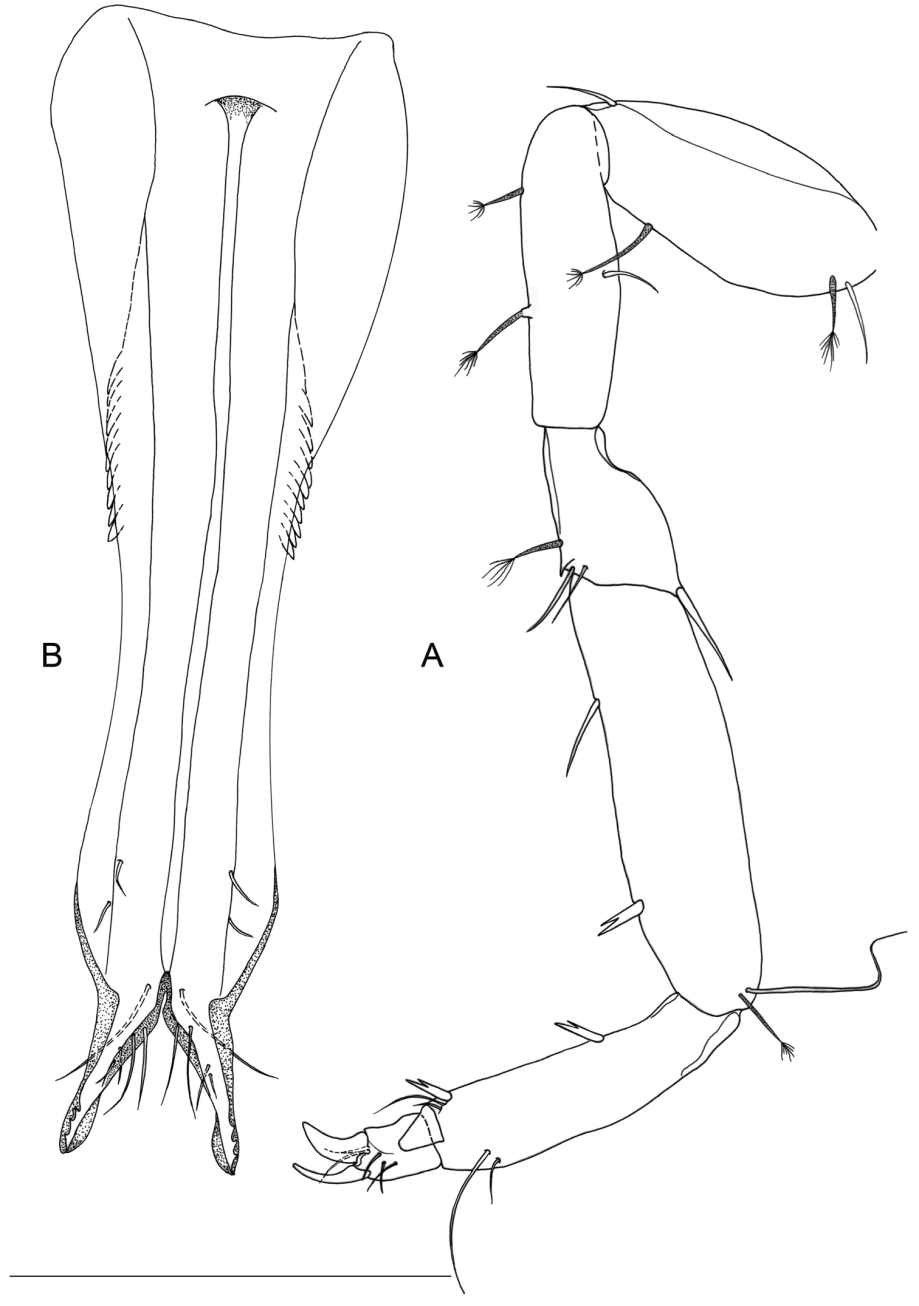
*Lepidocharon priapus* gen. n., sp. n. (♂ holotype). **A** pereiopod 7 **B** pleopod 1 (scale bar: 0.1 mm).

Pereiopod 1 (Figs 2E, 4E): coxal plate hardly discernible, basis slightly enlarged, relatively short in comparison to length of same segment of pereiopods 2–7, with 3 short setae, one of which is a penicillate seta; 2 opposite setae on ischium, transformed in sensorial penicillate setae; merus trapezoidal, shorter than all other articles, bearing 4 long and large setae on mesial and lateral apical margins; carpus longer than merus bearing 2 opposite long slender setae; mesiodistal margin with spinule row; propodus longer and slender than merus, ending with a small sclerite (Fig. 2E), with mesial hyaline lamella, bearing 1 bifid robust seta and 5 slender setae; dactylus with 6 slender sensorial setae, 3 of which inserted on surface of dactylus at base of insertion of longer claw, 2 surface setae inserted at basis of shorter claw, 1 seta on mesial distal margin of dactylus. Pereiopods 2–7 with strong dactylar claws with rounded tip. Morphology, relative length of pereiopodal segments and their armature apparently identical. Pereiopods 2–7 (pereiopod 7 figured; Fig. 5A) with coxal plate hardly discernible, basis slender than in pereiopod 1, bearing 4 setae, 2 of which penicillate, and 2 not transformed slender setae; ischium longer than in pereiopod 1, rectangular in shape, bearing 3 setae, 2 of which penicillate; merus shorter than articles, trapezoidal, and stouter than in pereiopod 1, bearing 1 sensorial seta on mesial margin, and 2 robust spiniform setae on apical mesial and lateral margins, respectively; a pointed protrusion discernible on mesial margin in subapical position accompanied by 1 thin seta; carpus longest; longer than both merus and propodus, bearing 4 elements; 1 proximal mesial simple seta and 1 bifid spine; 2 lateral setae located on apical lateral margin, 1 of which thin and long, the latter transformed in a penicillate seta; propodus long and slender than carpus, ending with elongate sclerite (Fig. 2F), bearing 2 bifid stout spines on mesial margin and 4 simple thin setae of different lengths; dactylus ending with two stout claws subequal in length, armed with 5 thin setae likely with sensorial function, 3 of which inserted on surface of dactylus at base of the insertion of longer claw, 2 surface setae inserted at basis of shorter claw.

**Figure 6.**
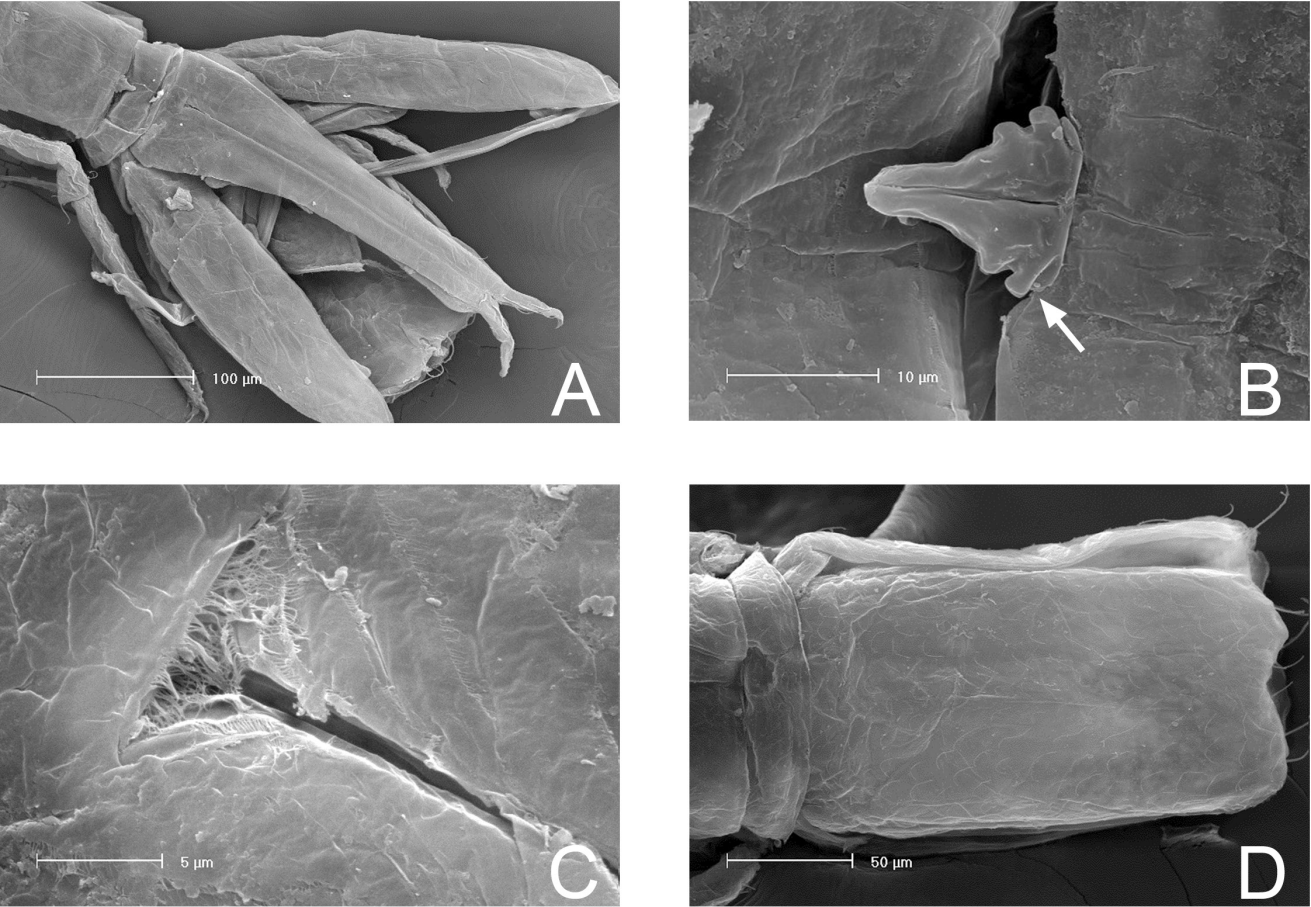
SEM micrographs of *Lepidocharon priapus* gen. n., sp. n. **A, B, C** ♂ paratype. **A** general view of free pleonite (ventral view), and pleopod 1 and 2 **B** penial papillae with ventral groove (arrowed) **C** pleopod 1, detail of the anterior proximal opening to the mesial channel **D** ♀ paratype, operculum.

Pleonite 0.29 as long as and 0.84 as wide as pereionite 7 (Figs 1A, 6A), small, narrower and shorter than pereionites and pleotelson; well discernible on dorsal and ventral views, partially covered by pereionite 7 on ventral view (Fig. 6A).

Penial papillae, as for the genus, coalescent and located at the middle of the free posterodistal margin of pereionite 7, with undulated free outer margins and a medial channel (Fig. 6A–B).

Pleotelson 1.37 as long as maximal width (Figs 8D, 15A), longer than wide (length/width ratio: from 1.75 to 2.00, n = 5). Dorsal side with semicircular thickening, as dorsal and ventral body surfaces; with 4 dorsal setae, arranged in two pairs; lateral margins each bearing 3 slender setae. Pleotelson distal margin with 12 marginal setae, inserted in apical or subapical position; 2 of them are penicillate setae.

Male pleopods 1 elongate and slender, fused proximally, with sperm tube medially with an anterior opening ornamented by small spines (Figs 5B, 6A, C), approximately 3.3 as long as maximum width (measured at widest section of proximal part of pleopod). Proximal part of pleopod large and gradually tapering at distal part, bordered by paired rows of 10 imbricate scale-like elements on posterior surface. Middle part of pleopod with free lateral margins smooth, parallel, and slender, ending with convex rounded margins, tapering apically, with paired well-developed distal protrusions, each ending with rounded apex, a hyaline membrane, crenulated on lateral margin, and smooth on mesial margin. Stylet-guiding groove parallel to lateral margins of pleopod, and folded by hyaline lamella, sclerotized in terminal part on both lateral and mesial sides of distal part of pleopod. Distal part of pleopod with 7 setae.

**Figure 7.**
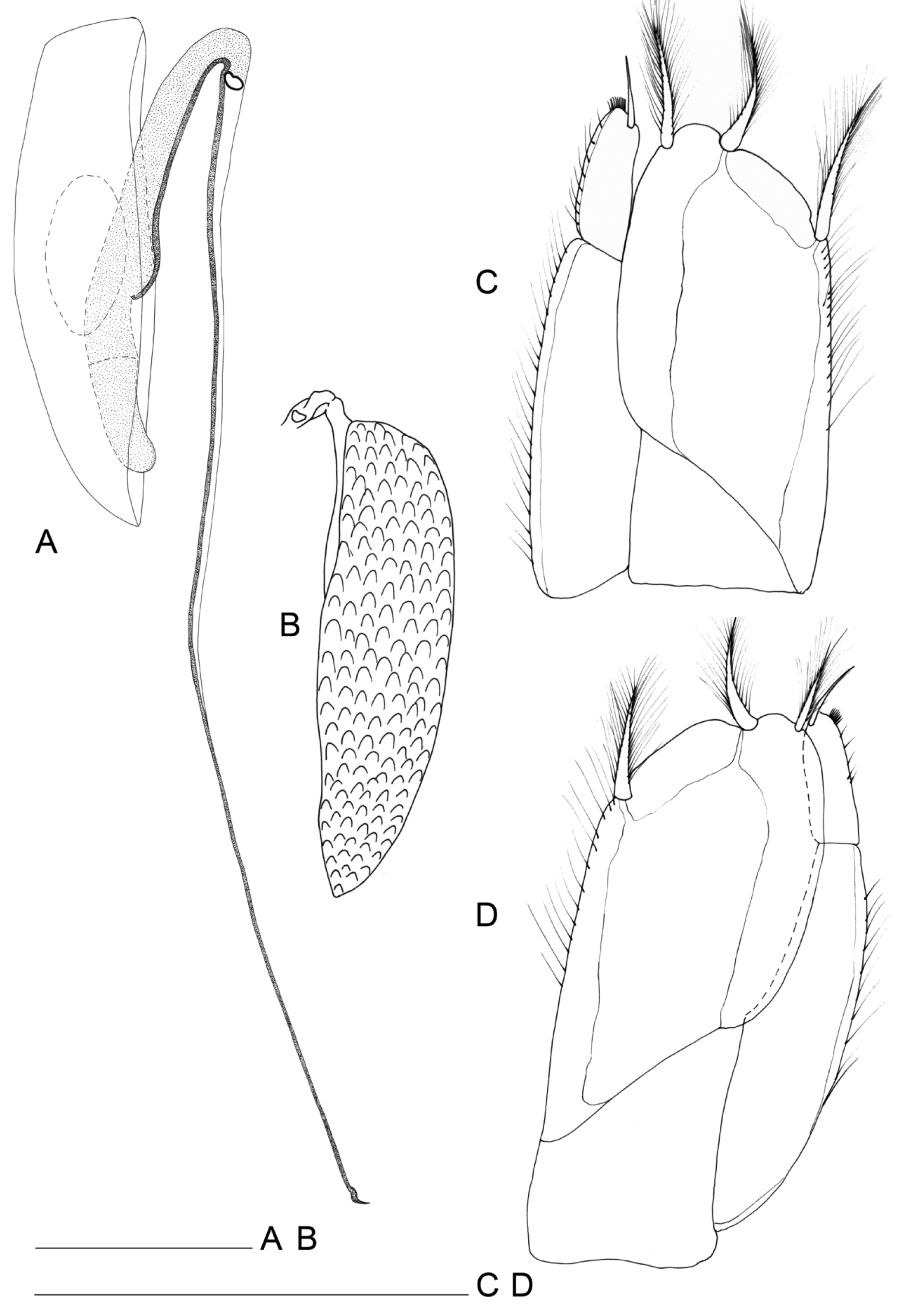
*Lepidocharon priapus* gen. n., sp. n. **A, C, D** ♂ holotype. **A** pleopod 2 **B** ♂ paratype, pleopod 2, protopod, **C** right pleopod 3 **D** left pleopod 3 (scale bar: 0.1 mm).

Male pleopod 2 (Fig. 7A): protopod elongate, sub-rectangular at its proximal part, and with rounded mediodistal corner; surface of protopod with semicircular thickening (Fig. 7B), exopod extruding partially from distal part of protopod, *appendix masculina* (endopod) extraordinarily long, more than 3 times (3.0–3.3; n = 4) the protopod length, ending with a long stylet with a sclerotized rib that runs along its entire length; terminal part with inflated lateral margins tapering to an acute tip. Stylet quite overreaching distal part of protopod, and reaching in length distal part of uropods.

Pleopod 3 (Fig. 7C, D) with endopod bearing 1 apical, 1 mesial subapical and 1 lateral plumose setae; exopod 2-segmented, with setulose hyaline lamella on lateral margin; exopod 1 elongate, 2.8 times longer than exopod 2, the latter ending with short simple seta.

**Figure 8.**
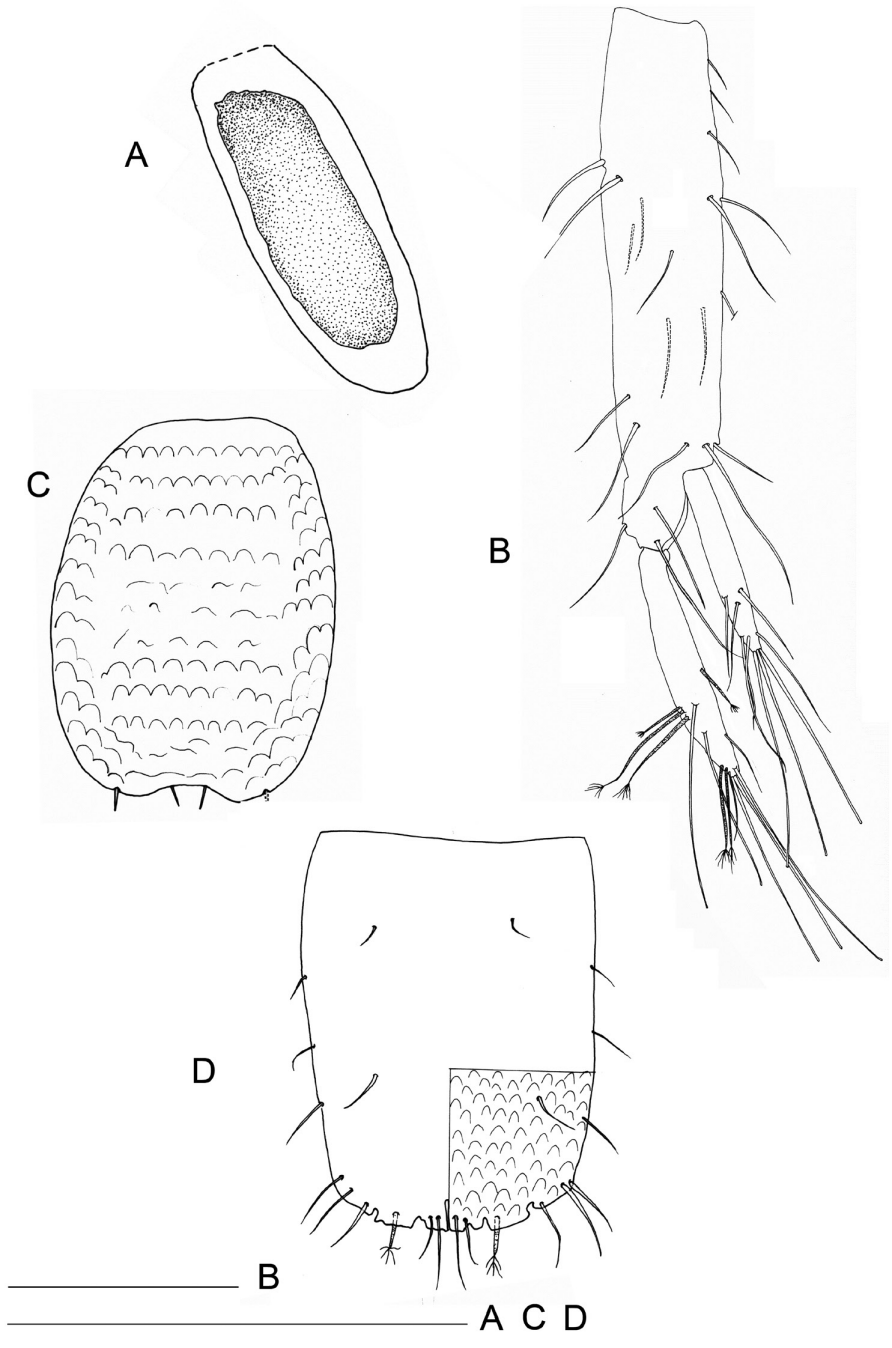
*Lepidocharon priapus* gen. n., sp. n. **A, B, D** ♂ holotype. **A** pleopod 4 **B** uropod **C** ♀ paratype, pleopod 2 (operculum) **D** pleotelson (scale bars: 0.1 mm).

Pleopod 4 (Fig. 8A) rudimentary, ellipsoidal, uniramous.

Uropods long and slender (Fig. 8B), approximately two times longer than pleotelson. Protopod long, two times longer than endopod. Exopod and endopod long and slender; endopod longer than exopod, the latter inserted in subapical position on protopod. Mesial margin of protopod armed with 5 setae; lateral margin with 8 setae, 8 surface setae, 4 of which are slender and located on ventral surface, remaining setae on dorsal surface large. Endopod with long setae on distal third, 6 of which are penicillate setae, remaining 7 setae slender, simple, with rounded tip. Exopod armed with 9 setae on distal third, all of which slender; apicalmost setae longest.

##### Female.

Body length generally similar to male (Fig. 15A). Body length measured from tip of cephalon to end of pleotelson from 0.9 to 1.4 mm. (n = 3). Length/width ratio: ~8.5. Female operculum elongate (Figs 6D, 8C), sub-rectangular in shape, with parallel lateral margins, proximal margin straight; distal part medially incised, bearing 2 close-set short medial setae and 2 setae in apical lateral position. Operculum surface with semicircular thickening (Fig. 6D).

##### Remarks.

Detailed comparison between the two species is given in the remarks for *Lepidocharon lizardensis* sp. n.

#### Lepidocharon lizardensis

Taxon classificationAnimaliaIsopodaLepidocharontidae

Galassi & Bruce sp. n.

http://zoobank.org/CE092992-8ECA-4891-B2A7-1396F8263D3F

**Figure 9.**
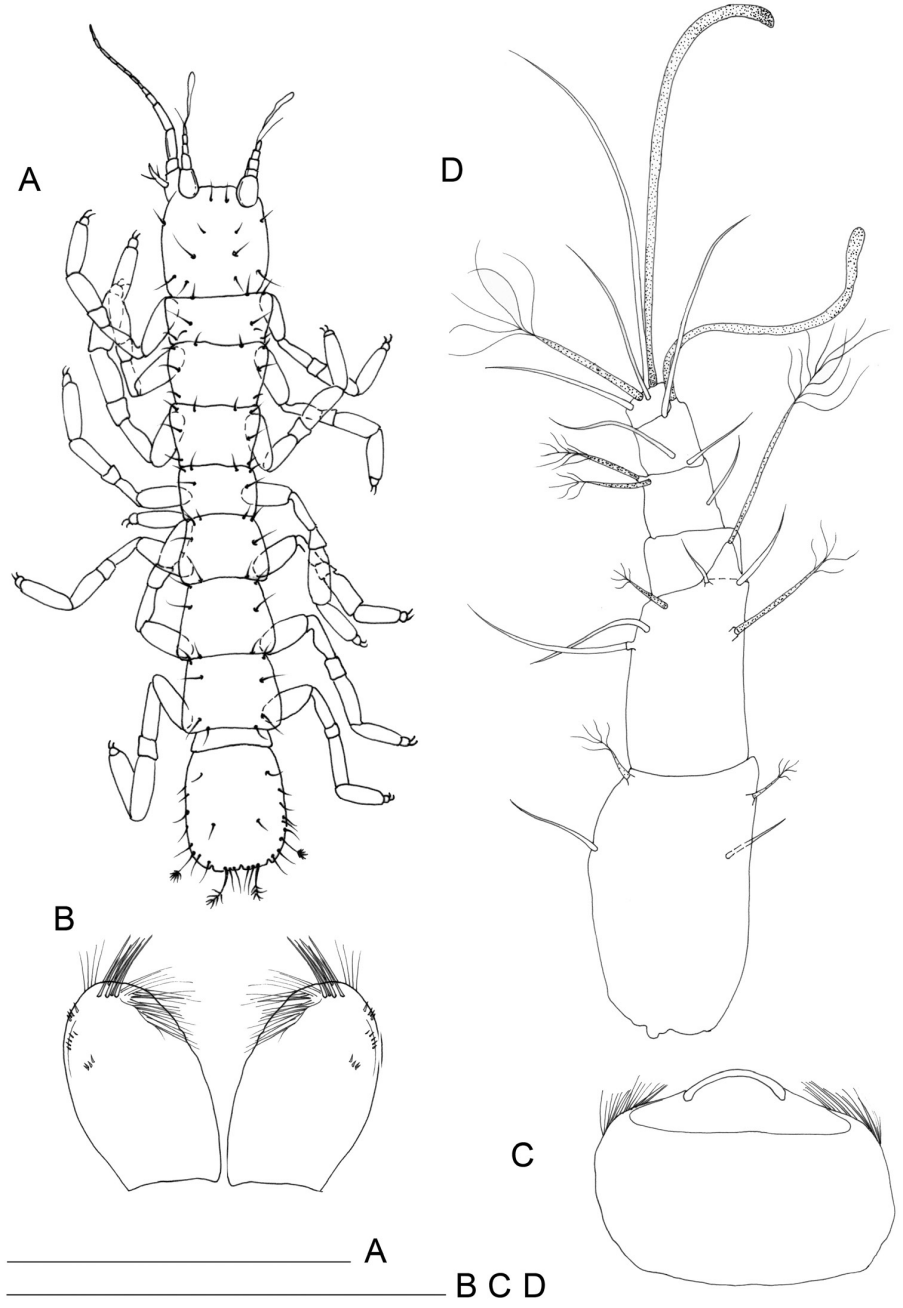
*Lepidocharon lizardensis* gen. n., sp. n. ♂ holotype. **A** habitus **B** paragnaths **C** labrum **D** antennula (scale bars: **A** 0.5 mm; **B, C, D** 0.1 mm).

**Figure 10.**
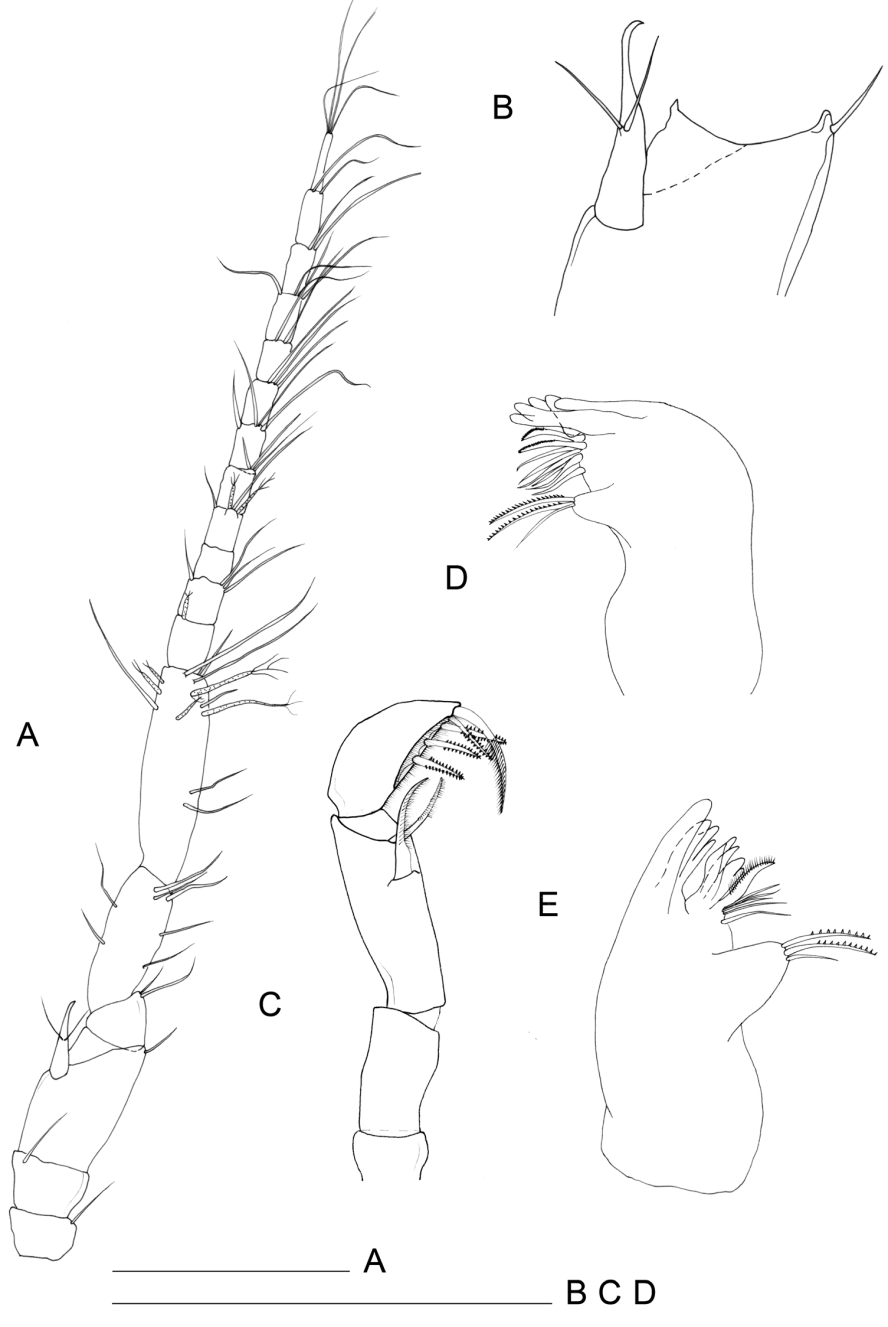
*Lepidocharon lizardensis* gen. n., sp. n. ♂ holotype. **A** antenna **B** detail of the antennal scale **C** mandibular palp **D** left mandible **E** right mandible (scale bars: 0.1 mm).

**Figure 11.**
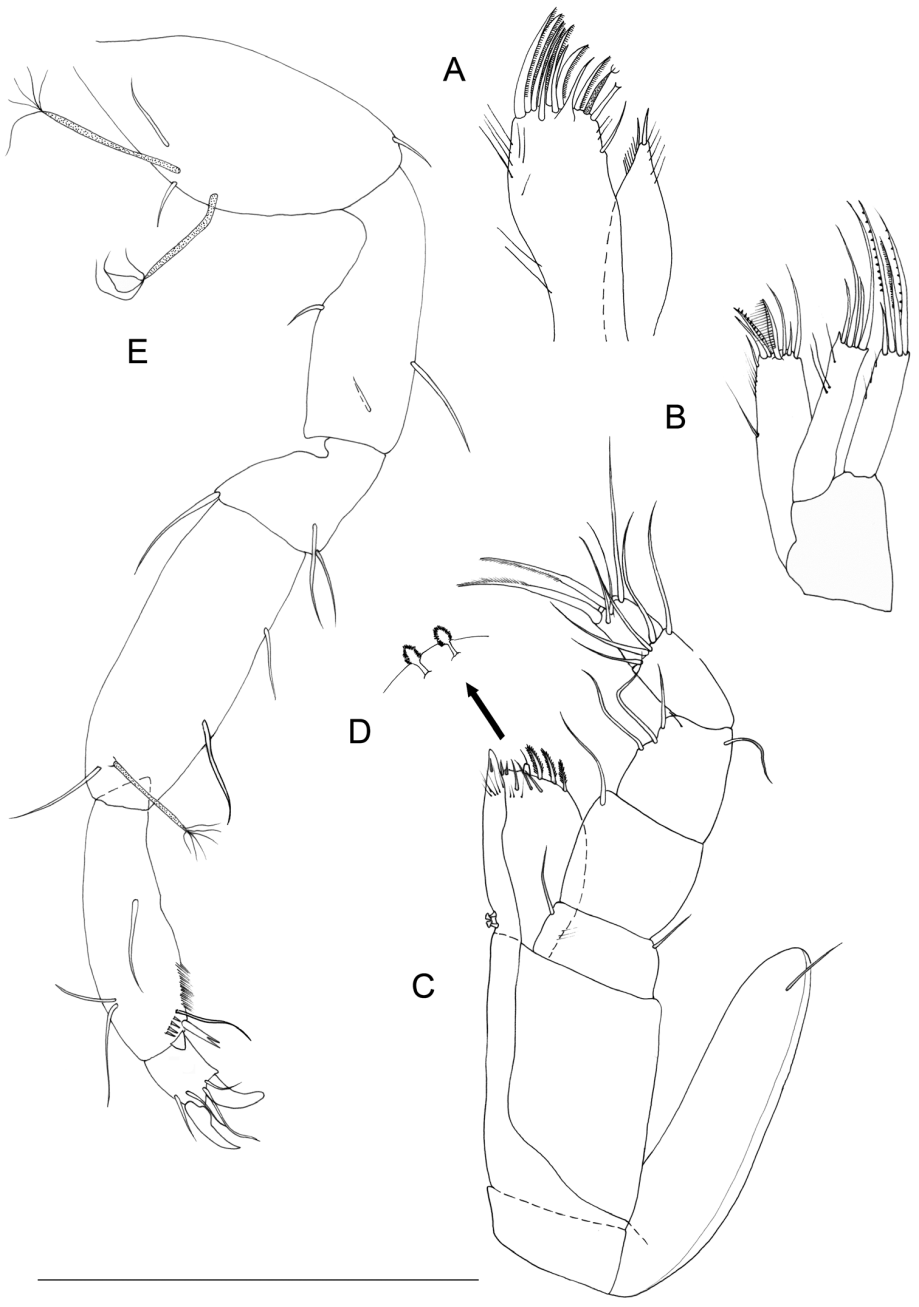
*Lepidocharon lizardensis* gen. n., sp. n. ♂ holotype. **A** maxillula **B** maxilla **C** maxilliped **D** maxilliped, detail of the subapical setae morphology of the endite **E** pereiopod 1 (scale bar: 0.1 mm).

**Figure 12.**
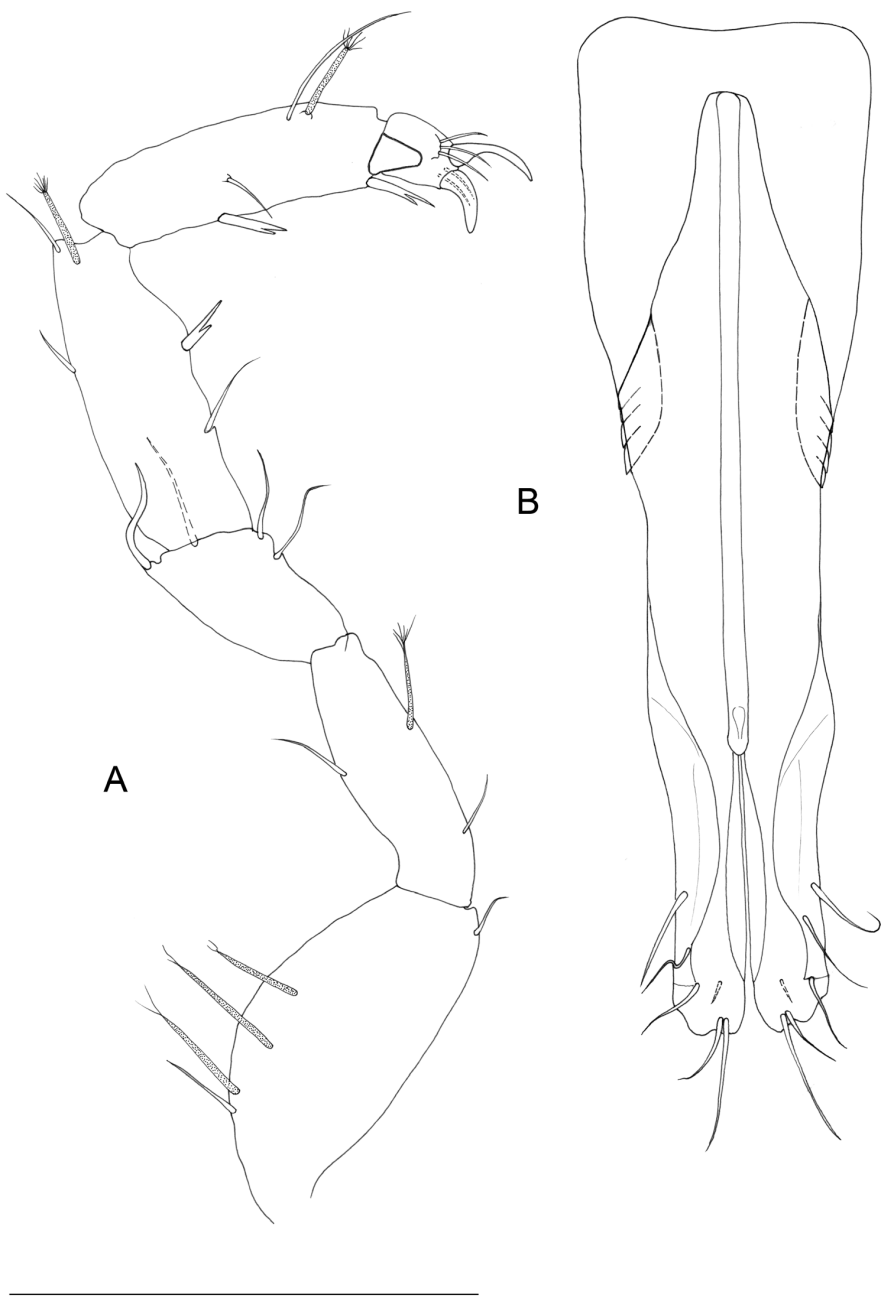
*Lepidocharon lizardensis* gen. n., sp. n. ♂ holotype. **A** pereiopod 7 **B** pleopod 1 (scale bar: 0.1 mm).

**Figure 13.**
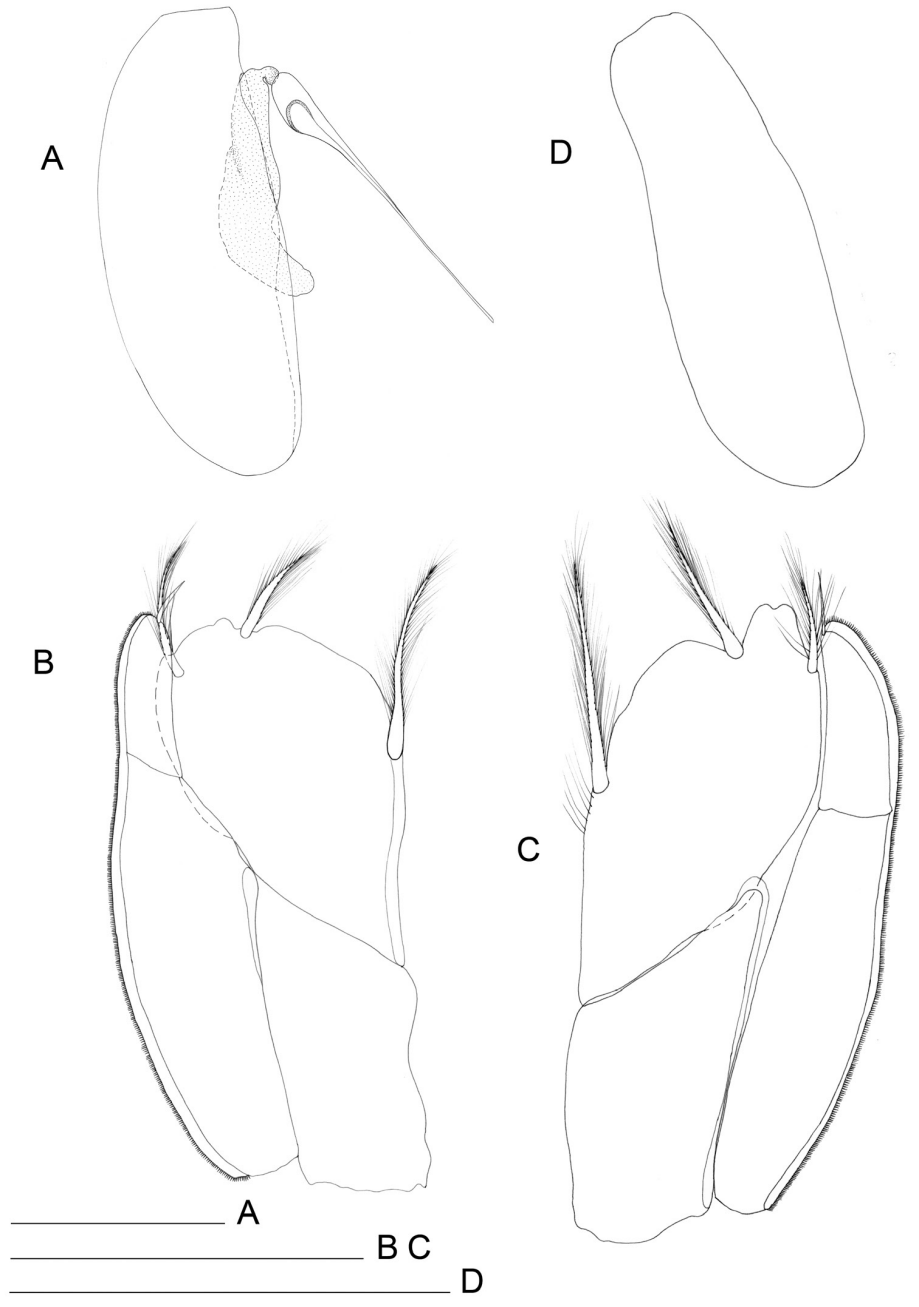
*Lepidocharon lizardensis* gen. n., sp. n. **A, C** ♂ holotype. **A** pleopod 2 **B** ♂ paratype, right pleopod 3 **C** left pleopod 3 **D** ♂ paratype, pleopod 4 (scale bars: 0.1 mm).

Figures 9 , 10 , 11 , 12 , 13 , 15B

##### Material examined.

*Holotype* here designated. Adult ♂ (1.1 mm), completely dissected and mounted in polyvinyl lactophenol on one slide, 17 February 2009; coll. N.L. Bruce and M. Błażewicz-Paszkowycz (MTQ W28330).

Type-locality: Australia, Lizard Island, off Coconut Beach, 14.68441°S, 145.47197°E, reef front, sand adjacent to bommies, 4 m, stn Liz 09-09A.

*Paratypes.* 1 ♂ (0.9 mm), 1 ♀ (1.2 mm) completely dissected and mounted in polyvinyl lactophenol, same data as holotype (all MTQ W28331).

##### Etymology.

The epithet *lizardensis* is after the type locality, Lizard Island, northern Great Barrier Reef, Queensland.

##### Description of male.

Body length measured from tip of cephalon to end of pleotelson 0.9–1.1 mm (n = 2). Body dorso-ventrally flattened, stocky, small-sized, about 6.5 times longer than wide (Figs 9A, 15B). Cephalon as long as wide (length/width ratio: ~1), as large as pereionites, lateral margins sub-parallel; anterior margin not projecting, rostrum absent. Dorsal surface of cephalon, pereionites and pleotelson (except free pleonite) ornamented with dorsal setules, arranged symmetrically and in pairs (Fig. 9A).

Pereionites 1–7 subequal in width (Fig. 9A); pereionites 1–3 with anterolateral margins of tergites only slightly protruding; pereionite 4 rectangular, without protrusions, pereionites 5–7 with posterolateral margins of tergites slightly protruded. Pereiopods inserted on lateral margins of tergites, visible in dorsal view (Fig. 9A); coxal plates rudimentary, incorporated to sternites.

Paragnaths (Fig. 9B) consisting of 2 large rounded lobes, deeply incised on medial side, ornamented with long setules on free mesial margins; thin simple setae are accompanied by small setules. Lateral margins with three short spinule rows. Labrum ovoid (Fig. 9C), with free anterior margin convex and medially thickened, with pair of thin scale-setae inserted symmetrically on the outermost sides of the free distal margin.

Antennula (Fig. 9D) composed of 6 articles; article 1 broadest, 1.6 as long as wide, directed anteriorly, with 2 simple and 2 penicillate setae; article 2 narrow, 1.6 as long as wide, 0.6 as wide and 0.7 as long as article 1, with 5 setae inserted at distal third of article, two of which penicillate; 1 long sensorial aesthetasc-like seta inserted on lateral protrusion, accompanied by a short and slender simple seta on its basis; article 3 unarmed; article 4 with 1 lateral simple seta and 2 penicillate setae inserted in apical position; article 5 slightly shorter (0.86) than article 4, bearing 1 long aesthetasc and 1 simple long slender seta at base of aesthetasc-bearing protrusion; 2 simple setae inserted at surface of article; article 6 very short, clearly articulated with article 5, bearing 2 subapical lateral setae, one of which aesthetasc-like penicillate seta, and 1 long apical seta close to 1 long aesthetasc and 1 subapical slender seta between them.

Antenna (Fig. 10A–B): with 6 podomeres, articles 1 and 2 short, article 1 with mesial seta; article 2 with short lateral seta; article 3 robust, with mesial apical seta and long exopod overreaching segment 4, candle-flame shaped and bearing 2 short and slender setae inserted at middle of exopod; article 4 stout and curved with 2 apical mesial setae; articles 5 and 6 slender, article 6 longest, bearing respectively 7 and 12 armature elements, 5 of which transformed in penicillate setae with apical tuft; flagellum composed of 12 articles, all flagellar articles with setae on distal margins, most setae simple; some on flagellar articles 1 and 4 are penicillate setae.

Mandible. Palp (Fig. 10C) on short cuticular projection. Palp article 1 naked, article 2 longest, about 2.5 times as long as wide, with 2 pinnate robust setae laterally, their insertion more or less coalescent with article; article 3 curved laterally, with 4 spinulose and 1 apicalmost unipinnate setae, distalmost longest and stout; 2 setule rows on lateral margin of article. Left mandible (Fig. 10D): incisor with 3 cusps; *lacinia mobilis* with 2 teeth; molar process with 2 long unipinnate spines and 1 short smooth seta. Between *lacinia mobilis* and molar process 2 transformed crested setae are present; 6 slender, long simple setae complement total pattern of 8 elements. Right mandible (Fig. 10E) incisor with 8 robust cusps; molar process with 3 apical spines, 2 of which unipinnate and robust, proximalmost naked and shorter; between incisor and molar process 6 spines are inserted, proximalmost curved, robust and uniserrate; remaining spines simple.

Maxillula (Fig. 11A) with slender mesial lobe tapering at distal part bearing 1 short apical seta accompanied by secondary subapical short seta and hair-like setules. Lateral lobe sub-rectangular in shape, bearing scale-setae on both lateral and mesial margins. Apical setation composed of 13 elements; 1 mesial slender seta, 1 mesialmost apical seta with bifid tip; 1 penicillate seta, 7 uniserrate setae, and 3 subapical surface simple setae.

Maxilla (Fig. 11B) mesial ramus with 8 setae, 2 naked setae on mesial margin, 6 apical setae, mesialmost naked, 1 uniserrate, third seta comb-teeth shaped, unipinnate, ornamented by fine setules regularly spaced and parallel to one another; remaining 3 setae simple. Lateral rami close-set, each bearing 4 setae of different lengths; lateralmost ramus with 3 spinulose and 1 short mesial setae.

Maxilliped (Fig. 11C–D) palp robust and curved mesially; article 1 sub-rectangular in shape, bearing 1 lateral and 1 mesial short setae; article 2 robust, bearing 1 apical mesial seta; article 3 as long as article 2, with 1 lateral apical and 3 mesial setae inserted on mesial margin; article 4 curved inwards, slender, with 1 lateral and 4 apical setae inserted at boundary line between article 4 and 5; article 5 short and narrow, with 2 apical and 3 subapical setae, of which 3 are simple slender setae of different lengths and 2 are robust and large, stiff pectinate setae. Endite almost reaching end of palp article 2; mesial margin ending in pointed protrusion, with numerous hair-like setules and 2 coupling hooks mesially; apical free distal margin with 3 spine-like, serrate setae, 1 simple non-tapered seta, and 2 surface fan setae; epipod ovoidal, overreaching distal part of palp article 1, bearing 1 subapical short, minute seta (this seta not found in other specimens).

Pereiopod 1 (Fig. 11E) coxal plate hardly discernible; basis slightly enlarged, shorter than that of P2–P7, with 2 short setae on mesial surface and 1 short simple seta on distolateral margin, and 2 penicillate setae; 2 opposite setae on ischium, one of which longer, a tubular sensorial seta on surface; merus shorter than other articles, trapezoidal, bearing 3 long setae; carpus slender and longest, mesial margin with 2 thin setae; distolateral margin with 1 simple slender seta and 1 penicillate seta; propodus slender than carpus, ending with a small elongate sclerite, with 2 mesial slender setae and 1 bifid spiniform seta; spinule row on free distal mesial margin; 3 simple setae on anterior surface; a spinule row at insertion of bifid spine; dactylus with 4 thin sensorial setae, inserted in pairs at base of each claw. Pereiopods 2–7 (pereiopod 7 figured; Fig. 12A) with coxal plate rudimentary and incorporated to the sternite, basis slender than in pereiopod 1, bearing 5 setae, three of which penicillate and two slender simple setae; ischium slightly longer and slender than in pereiopod 1, rectangular in shape, bearing 3 setae, 1 of which transformed in penicillate seta; merus shorter than all other leg segments, trapezoidal, and slightly longer and slender than in pereiopod 1, bearing 2 setae on mesial margin, 1 robust seta on apical lateral margin and 1 slender simple seta on surface at boundary between merus and carpus; carpus almost as long as propodus; robust, bearing 1 simple thin seta and a bifid spine along mesial margin, and 2 short thin setae on lateral margin, a penicillate seta inserted close to apicalmost lateral seta; propodus slender than carpus, with small elongate sclerite, bearing 2 bifid stout spine-like setae on mesial margin, 2 surface simple setae of different lengths and a surface penicillate seta at distal third of article; dactylus ending with 2 strong claws with rounded tip, slightly subequal in length, lateral claw slender, mesial stouter and shorter; dactylus armed with 5 thin setae likely with sensorial function, 3 of which inserted at surface of article, at base of insertion of the longer claw, 2 surface setae inserted at basis of shorter and stouter claw.

**Figure 14.**
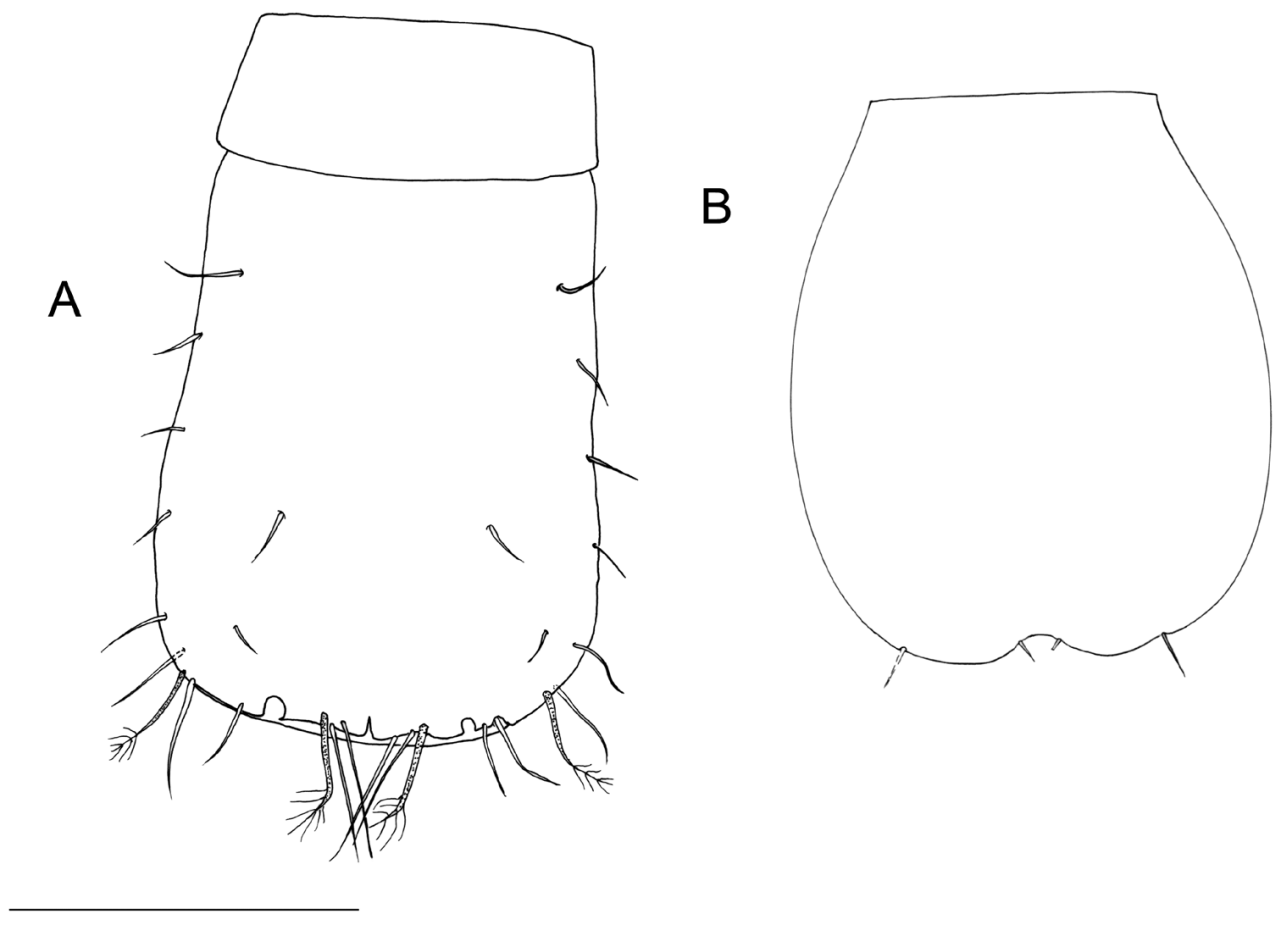
*Lepidocharon lizardensis* gen. n., sp. n. **A** ♂ holotype, pleonite free and pleotelson **B** ♀ paratype, pleopod 2 (operculum) (scale bar: 0.1 mm).

**Figure 15.**
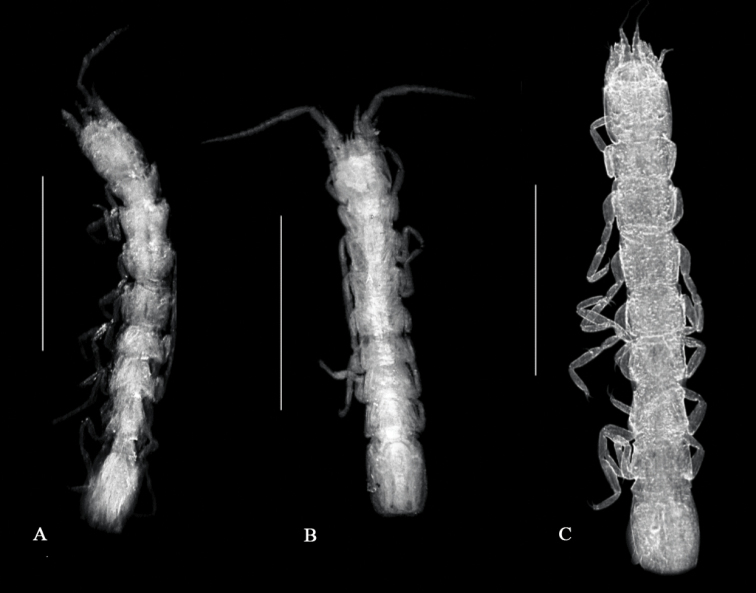
Stereomicroscope images of the habitus of **A** *Lepidocharon priapus* gen. n., sp. n. ♀ paratype **B** *Lepidocharon lizardensis* gen. n., sp. n. ♀ paratype **C** *Microcharon reginae* Dole and Coineau, 1987 ♀ topotype, showing the different morphology of the pereionites, the topology of pereiopods, and the different degree of development of the single pleonite free (scale bars: 0.5 mm).

Pleonite length 0.27 pereionite 7 length, width 0.86 pereionite 7 width (Figs 9A, 14A, 15B).

Pleotelson longer than wide (Fig. 14A, 15B) (length/width ratio: from 1.33 to 2.00, n = 2). Dorsal side with 6 setae, a pair located on proximal part of pleotelson, other 2 pairs of setae arranged in close-set two pairs, both pairs located on distal third of pleotelson. Lateral margins bearing 3 slender setae at each side; 16 setae bordering free distal margin of pleotelson, inserted in apical or subapical position; 4 of them are penicillate setae.

Male pleopods 1 (Fig. 12B) elongate, coalescent in proximal part, with sperm tube medially, length about 4.5 times longer than maximum width (measured at widest section of proximal part of pleopod). Proximal part of pleopod large and gradually tapering at its distal part, bordered by paired rows of 4 scale–like elements. Middle part of pleopod with free distal margins smooth, parallel and slender, ending with slightly inflated sub-distal rims, tapering in apical part with paired well-developed sub-rounded bilobate tips. Stylet-guiding groove folded by hyaline lamella, running parallel to lateral margins of pleopod, only slightly sclerotized and ending with a transversal straight margin. Set of distal setae composed by 6 elements only.

Male pleopod 2 (Fig. 13A): protopod elongate, sub-rectangular at proximal part, with rounded mediodistal corner; exopod protruding from protopod at middle of mesial margin, *appendix masculina* (endopod) stylet ending with skewed apex, opening oblique; stylet short, shorter than and not reaching distal part of protopod.

Pleopod 3 (Fig. 13B–C) exopod bearing 1 apical plumose seta, 1 lateral subapical medial long plumose seta and 1 subapical mesial plumose seta. Between mesial and apical setae exopod is protruded in rounded or bilobate lobes (Fig. 13B–C); endopod with setulose hyaline lamella on mesial margin bordered by fine setule row; endopod 1 elongate, about 2.2 longer than endopod 2, the latter ending with short simple subapical seta.

Pleopod 4 (Fig. 13D) rudimentary, ellipsoidal, uniramous.

Uropods unknown.

##### Female.

Body length approximately as in male. Body length measured from tip of cephalon to end of pleotelson 1.2 mm. No sexual dimorphism observed in body morphology, cephalic appendages and pereiopods. Female operculum (pleopod 2) elongate (Fig. 14B), sub-ovoid, with rounded lateral margins, proximal margin straight; distal margin with medial incision, with 2 close-set short medial setae very close to medial incision and 2 longer apical setae in lateral position. Operculum surface smooth. Uropods unknown.

##### Remarks.

*Lepidocharon priapus* and *Lepidocharon lizardensis* differ from each other in several characters: 1) the morphology of the antennal scale (blade-knife shaped in *Lepidocharon priapus* *vs.* candle-flame shaped in *Lepidocharon lizardensis*); 2) the slender body with different degree of protrusion of the pereionites 1–3 and 5–7 (markedly protruded in *Lepidocharon priapus vs.* stouter and less protruded in *Lepidocharon lizardensis*); 3) the different shape of the male pleopod 1 (with strongly protruded and pointed apical lobes and sclerotized hyaline lamella in *Lepidocharon priapus* vs. sub-rounded and undulated apical lobes and a tiny hyaline lamella in *Lepidocharon lizardensis*); 4) the setal complement of the male pleopod 1 (7 in *Lepidocharon priapus* vs. 6 in *Lepidocharon lizardensis*); 5) the morphology of the male pleopod 2 (sub-rectangular and long protopod with endopodal stylet extraordinarily long, reaching the tips of the uropods in *Lepidocharon priapus* vs. ovoidal protopod with a short stylet, not reaching the distal part of the protopod in *Lepidocharon lizardensis*); 6) female operculum sub-rectangular in shape, with straight lateral margins in *Lepidocharon priapus* vs. oval, with convex lateral margins in *Lepidocharon lizardensis*); 7) a rounded pleotelson in *Lepidocharon priapus* (sub-truncate in *Lepidocharon lizardensis*), and 8) body surface with visible semicircular thickening in *Lepidocharon priapus* (smooth in *Lepidocharon lizardensis*).

## Discussion

The family Microparasellidae has been provisionally assigned to the asellote superfamily Janiroidea Sars, 1897. Its monophyletic status was debated since the interpretation of most character states in microparasellids is still doubtful, if not questionable (Bocquet and Lévi 1955, Wolf 1962, Cvetkov 1968, Wilson 1987, Wilson and Wägele 1994) in recognizing family rank for the Microparasellidae. Its monophyletic status had been hypothesised by Coineau and Schmidt (1979) and Coineau (1986, 1994). Since the original diagnosis given by Karaman (1933) (without providing the family name) and the provisional diagnosis given by Wilson and Wägele (1994) in their review of the family Janiridae, it had become clear that several diagnostic characters are weak and others have not been considered in detail (Albuquerque et al. 2014), and both the Janiridae and Microparasellidae were placed as *incertae sedis* (*sedis mutabilis* according to Wilson 1987: page 776) in the suborder Asellota.

Wilson and Wägele (1994) produced a critical review of the genera attributable to the former family Microparasellidae, arguing the definitive exclusion of the genus *Protocharon* Chappuis, Delamare Deboutteville & Paulian, 1956 from this family on the basis of the closest similarity, they claimed, between *Protocharon* and *Iais* Bovallius, 1886, with *Protocharon antarctica* Chappuis, 1958 definitively moved to the janirid genus *Iais* (Wilson and Wägele 1994: page 700), and *Protocharon arenicola* Chappuis, Delamare Deboutteville & Paulian, 1956 being the only species attributable to the now monotypic genus *Protocharon*.

Similar arguments had been anticipated by Wolf (1962) who highlighted that blind and colourless species occur in almost all the families of the suborder Asellota; species belonging to the same genus may or may not have eyes. Anophtalmy is a common convergence to lightless or extreme low-light habitats, such as the deep-sea, groundwater habitats, mud, crevices, and as such is useless in assessing phylogenetic relationships. For instance, several Janiridae are eyeless and small-sized (e.g., species of *Jaera* Leach, 1814, *Heterias* Richardson, 1904, *Austrofilus* Hodgson, 1910, *Caecijaera* Menzies, 1951, *Caecianiropsis* Menzies & Pettit, 1956), and have neither prehensile nor subchelate pereiopod 1, as have all the presently known species assigned to both Microparasellidae and Lepidocharontidae.

Among the genera assigned to the former family Microparasellidae, the genus *Angeliera* uniquely has a 7-segmented antennula, antennal scale absent; the maxilliped palp 4-segmented, without apical stiff pectinate setae; most pereiopods (variable among species of the genus) with three claws; pereiopods 1–4 and 6–7 subsimilar, with pereiopod 5 sexually dimorphic, in the male being subchelate and stout, with carpus transformed; male pleopod 1 is short and sub-quadrate and the penial processes are paired, not coalescent, arising near the base of pereiopod 7 (i.e. laterally) (see for details Coineau 1968). The female operculum is shorter than pleotelson, deeply incised or concave on free distal margin, without apical setae, never reaching the free distal margin of the pleotelson. Furthermore in *Angeliera* the male pleopod 2 displays a short and truncate form, similar to that of the Vermectiadidae Just & Poore, 1992. The family Vermectiadidae Just & Poore, 1992 shows superficial similarities to the genus *Angeliera* but can immediately be distinguished by having three free pleonites, laminar pleopods, none of which form an operculum, as well as small eyes (Just and Poore 1992).

*Angeliera* lacks the molar process, the most derived condition found in the Janiroidea. Nevertheless, similar reductions of the molar process occur in the janiroid family Katianiridae Svavarsson, 1987, where it varies from being reduced to a single spine in *Katianira acarina* (Menzies, 1962) (Svavarsson 1987) to lost in *Katianira platyura* Shimomura & Akiyama, 2006 (Shimomura and Akiyama 2006: fig. 1D, E, page 578). Similar reductions occur also in members of other janiroid families (e.g. Nannoniscidae Hansen, 1916, Desmosomatidae G.O. Sars, 1897, Macrostylidae Hansen, 1916, Munnopsidae Lilljeborg, 1864) and are likely adaptive traits related to the trophic niche of the taxa. The fourth article of the maxillipedal palp in *Angeliera* has one apical seta only instead of the two distal stiff setae present in all the other members of the family. As the maxilliped is considered homologous to the pereiopods (for details see Thompson 2013), the condition showed by *Angeliera*, with only one apical seta, coalescent with the fourth article, is to be considered a derived condition in the Janiroidea.

Zemko and Kaiser (2012) discussed some affinities of the janiroidean family Thambematidae Stebbing, 1912, known from marine deep-sea habitats, and the former Microparasellidae, as previously claimed by Birstein (1961). The Thambematidae, on the basis of the similar body shape, was supposedly related to the Microparasellidae, even if the single free pleonite in Thambematidae is well-developed in comparison to the more reduced pleon of the Lepidocharontidae genera *Janinella* and *Lepidocharon*. Numerous characters separate the two families, including the morphology of the mandibular molar process, which is cylindrical in Thambematidae Stebbing, 1912 (*vs.* conical in Lepidocharontidae fam. n.), flat and prominently setose pereiopods (*vs.* slender), pereiopod 1 distinctly prehensile (*vs.* never prehensile neither subchelate in Microparasellidae and Lepidocharontidae fam. n.), male pleopod 1 with disto-lateral protrusions representing the extension of the transversal stylet guiding groove (only superficially resembling the general construction found in *Janinella* and in some Janiridae) and short, cylindrical uropodal sympod (*vs.* flattened) (Harrison 1987, Zemko and Kaiser 2012).

*A single free pleonite* is shared by the Janiridae, the Microparasellidae and the Lepidocharontidae fam. n., as well as by other phylogenetically distant families (e.g. Thambematidae Stebbing, 1912; Paramunnidae Vanhöffen, 1914; Urstylidae Riehl, Wilson & Malyutina, 2014). Members of the genus *Microcharon*, as observed in *Microcharon reginae* Dole & Coineau, 1987, possess a large free pleonite (Fig. 15C). Species of *Janinella* (Albuquerque et al. 2014) and *Lepidocharon* have a shorter and narrower pleonite.

*The single free pleonite* is even more reduced in the janirid *Microjaera anisopoda* Bocquet & Lévi, 1955, as highlighted by Bocquet and Lévi (1955). These authors supported the hypothesis of a strict relationship between *Microjaera* Bocquet & Levi, 1955 and the microparasellid genus *Microparasellus* and the lepidocharontid genus *Microcharon* on the basis of the elongate and slender body, the small size, and other unspecified characters (Bocquet and Lévi 1955: page 128). Conversely, the differences observed and listed by the same authors among their *Microjaera* and *Microparasellus*-*Microcharon* (and *Angeliera*) support only a remote affinity among these genera. Later, a second species of the genus, *Microjaera morii* Shimomura, 2005, was described, this species showing a highly reduced free pleonite, that is not discernible in dorsal view, and visible only on ventral view (Shimomura 2005: fig. 3F, page 118).

The relatively *large size of the free pleonite* in Microparasellidae (*Microparasellus*) has been considered a distinctive trait of the family by Wilson and Wägele (1994). After assessing the degree of development of this body somite, we observed that among the Lepidocharontidae, it is well-developed in *Microcharon*, being as large as pereionite 7. It is smaller and narrower in *Janinella* and *Lepidocharon*.

The *antennula* in the Lepidocharontidae is short, not sexually dimorphic, and composed of a maximum of 6 articles, this condition being derived in the Janiroidea. The segmental pattern of the antennula ranges from 6 to 5 in *Microcharon*, where the 5-segmented condition is shared by almost all the marine species of the genus, and the 6-segmented condition by fresh groundwater species, which likely retained the 6-segmented antennula of the ancient marine ancestor, in the more conservative and stable groundwater environment, according to Coineau (1986). Only the marine species *Microcharon monnioti* Bocquet, 1970 from the psammolittoral of Roscoff, France, possesses a 6-segmented antennula (even if the generic position of this species requires confirmation, N. Coineau, pers. comm.). The antennula is 5-segmented in *Janinella* and 6-segmented in *Lepidocharon*.

The *antennal exopod* (scale) is present in all the Lepidocharontidae genera. In general, the exopod is rudimentary in the Janiridae, and in members assigned to the Lepidocharontidae is ovoid and small, as in *Microcharon*, the most reduced condition being found in *Janinella* (Albuquerque et al. 2014) with a reduced scale, not reaching the tip of the third podomere. Conversely, it is long in *Lepidocharon*, reaching the fourth podomere, being candle-flame or knife-blade shaped.

The *molar process of the mandible* is always conical and reduced in the lepidocharontid genera *Microcharon*, *Janinella* and *Lepidocharon*.

In *Lepidocharon* the *mandibular palp* is inserted on short cuticular projection, as also observed in *Janiropsis* Sars, 1897 and *Janaira* Moreira & Pires, 1977 by Doti and Wilson (2010) and it is figured as a small well-defined article in *Trogloianiropsis lloberai* Jaume, 1995 (Jaume 1995: fig. 16, page 183). This condition is likely primitive in the Janiroidea, and seems to be retained by *Lepidocharon*.

The *distal article of the mandibular palp* bears from 5 to 3 robust setae, the latter status shared by the most derived groundwater species of *Microcharon*. The possession of 5 setae is shared by a few marine *Microcharon* species, when described, and the stygobiotic *Microcharon acherontis* Chappuis, 1942 is figured with 6 setae, a state not found in any other member of the genus.

In the *incertae sedis* genus *Angeliera*, the terminal segment of the mandibular palp has been described and figured without any seta or stiff setae, and some descriptions refer to the original description of *Angeliera phreaticola* Chappuis & Delamare Deboutteville, 1952 for which few details are available (Chappuis and Delamare 1952, Delamare Deboutteville 1960, Coineau and Rao 1972).

The *maxilliped* is 5-segmented in *Microcharon*, *Janinella* and *Lepidocharon*.

The genus *Angeliera* placed as *incertae sedis* in the Lepidocharontidae has a 4-segmented palp as other janiroid families, e.g., *Katianira* and *Natalianira* Kensley, 1984 (Katianiridae), raising questions about purported affinities between katianirids and the genus *Angeliera* (see Svavarsson 1987). The same author (Svavarsson 1987: page 717) rejected this assumption on the basis of the marked differences observed in *Katianira* and the former Microparasellidae in body shape, structure of the antenna and uropods.

The *maxilliped endite* has the same shape, recurrent in all members of the family, except for the genus *Angeliera*, where its distal part is swollen and ending with sinuous setae.

The *body morphology* is generally similar among members of the family Lepidocharontidae: pereional somites are rectangular in *Microcharon*, with a well-developed free pleonite, shorter and as wide as pereionite 7. *Janinella* and *Lepidocharon* have the anterior three pereionites markedly protruded anteriorly, the fourth almost rectangular in shape, and the last three pereionites protruded posteriorly. We assume that the morphology of the pereionites, together with the position of the pereiopods are related to a different way of locomotion of the species.

The *pereiopodal dactylus* has two unguli, showing a general tendency to be stout and subequal in length in marine species, and slender with the inner claw about ½ the length of the lateral claw in freshwater species.

The *morphology and armature of the female operculum* differs within the family. A character considered diagnostic for species distinction within the family is the number of apical setae bordering the female operculum. In *Microcharon* the highest variation occurs: from long, more than two times longer than wide, faintly incised female operculum in marine and freshwater species, bearing 4 or 2 apical setae (Galassi 1991), to rounded and unarmed in the most derived groundwater species (Coineau 1994).

The pleopod 3 has the *distal article of the endopod* either with 3 plumose setae or completely unarmed. The marine species always have these setae and they are consistently absent in all groundwater species.

*Angeliera* shows the most striking derived character states, suggesting a divergent position in relation to the remaining members of the family. *Janinella* shares with *Angeliera* the topology of the outwardly directed stylet-guiding groove of the male pleopod 1 on both pleopodal rami, a character that may be phylogenetically informative, and likely primitive in the family because it is shared by members of the closely related family Janiridae (e.g. *Jaera* Forsman, 1949, *Iais*, *Ectias* Richardson, 1906). *Microcharon* and *Lepidocharon* share stylet-guiding grooves folded by a hyaline lamella that run almost parallel to the free lateral margins of the distal parts of male pleopod 1, a likely derived organization and topology of the stylet-guiding groove if compared to that of *Janinella*. The stylet guiding-groove is in this case represented by a hyaline lamella, which holds the stylet of the pleopod 2 in a precise position. In the first case, as in *Jaera* (Wilson 1991: fig. 13.3, page 237), *Angeliera* and *Janinella*, the tip of the endopodal stylets of the second pleopod are directed outwards; conversely they run parallel to the lateral margins of pleopod 1 (Cvetkov 1968: fig. IV, page 118) in *Microcharon* and *Lepidocharon*, and the stylets are locked in a fixed position by lateral folding of the pleopods 1 (Cvetkov 1968: fig. IIA, page 115). We retain that these differences may have a phylogenetic significance, agreeing with Wilson (1991, 1994).

The penial papillae are differently organized in *Angeliera*: they are tubules which start from the insertion of the pereiopods on sternite 7 and converge at the midline of the sternite of the pereionite 7, maintaining their openings separate and not coalescent (Coineau 1971). In *Microcharon*, *Janinella* and *Lepidocharon*, there is no longer trace of paired penial papillae, as in most Janiroidea, and they are located at the postero-medial margin of the sternite, are very small, coalescent, even if still separated by a medial groove, likely the remnant of their paired origin. The few data available on *Microcharon* and *Janinella* show a medial position of the penial papillae (Coineau 1971); in *Lepidocharon* they are for the first time observed with the aid of scanning electron microscopy (Fig. 6B). According to Wilson (1987, 1991) little information is available on the precise function of the male pleopods 1 and 2 in the Janiroidea with regard to sperm transfer from the penial papillae (penes) to the female genitalia, being still unclear the role of the endopodal stylet of male pleopod 2, and the mating behaviour highly variable among the widely diversified asellote isopods. On this regard, it was argued that the different modes of copulation observed in the marine isopods may have played a key-role in their high diversification in marine and freshwater environments, allowing also the colonization of the deep-sea habitats (Wilson 1991).

The description of *Lepidocharon* Galassi & Bruce, gen. n. and the establishment of the Lepidocharontidae Galassi & Bruce, fam. n. shed new light on the diversity of the morphological body plans observable in the former family Microparasellidae, allowing for a better understanding of the phylogenetic relationships of the Lepidocharontidae fam. n. and the Microparasellidae, a family placed as *incertae sedis* within the superfamily Janiroidea Sars, 1897 (see Wilson 1994; Wilson and Wägele 1994).

## Conclusion

The small body size, soft cuticle, possession of the single reduced pleonite with laterally free margins, the simplification of the general structure and armature of the mandibles, the low diversification of setae morphology, the absence of pleopod 5, could be interpreted all together as derived character states, which may conversely be the result of homoplasy for adaptive convergence due to the interstitial life, or to life in microhabitats with reduced living space (Coineau 1986, 2000, Galassi et al. 1999).

In addition, the lack of morphological data in both historic and some recent contributions does not help in reconstructing an evolutionary scenario. Nonetheless, despite convergence, phylogenetic characters can be identified, and a greater degree of character resolution in family and genera definitions developed. Even if comprehensive data for all species are not available, it is evident that the current generic composition of the Microparasellidae could not be maintained. The separation of the Lepidocharontidae fam. n. and its constituent genera resolves the former paraphyletic family into two monophyletic families, which in future should enable a more clear understanding of the relationships of the two families to the Janiridae to be developed.

## Supplementary Material

XML Treatment for Lepidocharontidae

XML Treatment for Lepidocharon

XML Treatment for Lepidocharon priapus

XML Treatment for Lepidocharon lizardensis

XML Treatment for Microparasellidae

XML Treatment for Microparasellus

## Appendix 1

### Microparasellidae Karaman, 1934

The family name Microparasellidae was first formally proposed by Karaman (1934: page 44) although the family diagnosis was given the previous year (Karaman 1933: page 17) when describing the new species and new genus *Microparasellus puteanus* Karaman, 1933, with the accompanying statement “Microparasellus *n. fam., n. gen.*” At that time a type species was not designated for the genus *Microparasellus* (see Karaman 1933), nor one year later (see Karaman 1934). As previously stated, the family name was considered valid until 2000, and for this reason it is an available name as Microparasellidae Karaman, 1934.

#### Microparasellidae

Taxon classification AnimaliaIsopodaMicroparasellidae

Karaman, 1934

Microparasellidae Synonymy: Karaman 1934: 44.—Wolff 1962: 35.—Coineau 1986: 465.—Kensley and Schotte 1989: 90.—Wilson and Wägele 1994: 720.

##### Diagnosis of male.

Body slender, 4.0–6.0× long as wide, somites all subsimilar in width, head with acute or narrowly rounded rostrum. Lateral margins of head and pereionites convex, with cuticular scales; pleonite 1 laterally free; body without chromatophores. Eyes absent. Antennula flagellum with maximally 4 articles. Antennal scale absent. Antenna flagellum shorter than podomeres. Mandibular molar process distally pointed, without grinding surface, with several terminal setae. Pereiopods 1–7 not chelate or subchelate, almost identical in shape; all pereiopods with 2 dactylar claws. Pereiopods articulated latero-ventrally. Penial processes present, coalescent, with single medial opening on posterior margin of pereionite 7. Pleopods 1 and 2 not operculate in males; female pleopod 2 operculate; pleopod 3 endopod unarmed, exopod slender. Uropods uniramous, minute, stub-like, insertion ventro-terminal; single ramus, shorter than protopod. Anus terminal, not covered by pleopods.

##### Remarks.

The family Microparasellidae was ambiguously established by Karaman (1933) without formally stating family name anywhere in that publication. On the same occasion, the author described two new species of *Microparasellus*, namely *Microparasellus puteanus* Karaman, 1933 and *Microparasellus stygius* Karaman, 1933, but without designation of the type species for the genus *Microparasellus*, and without providing the name of the new family. One year later, Karaman (1934) formally proposed the family name Microparasellidae for the new family, also giving a family diagnosis and assigned two genera to the family: the then monotypic *Microparasellus* Karaman, 1933 (*Microparasellus puteanus*) and transferring *Microparasellus stygius* Karaman, 1933 to the new genus *Microcharon* Karaman, 1934 together with *Microcharon latus* Karaman, 1934 described in the same paper (Karaman 1934).

#### Microparasellus

Taxon classification AnimaliaIsopodaMicroparasellidae

Karaman, 1933

Microparasellus Karaman, 1933: 17; 1934: 44.—Wilson and Wägele 1994: 725.
Duslenia Lévi, 1950: 42.

##### Type species.

*Microparasellus puteanus* Karaman, 1933. Type locality: Skopje, Macedonia. Karaman (1934) formally described the family Microparasellidae, at the same time establishing the genus *Microcharon* Karaman, 1934. At that point Karaman restricted the genus *Microparasellus* to the single species *Microparasellus puteanus*. This in itself does not constitute a subsequent type species designation, and Karaman’s (1934) intention was clearly not to do that as he equally did not designate a type species for *Microcharon* (this was a common practice at that time).

##### Other species.

*Microparasellus libanicus* Chappuis & Delamare Deboutteville, 1954, Lebanon; *Microparasellus aloufi* Coineau, 1968, Lebanon; *Microparasellus hellenicus* Argano & Pesce, 1979, Greece.

##### Remarks.

The generic name *Microparasellus* established by Karaman (1933) is not an available name because, even though a diagnosis has been given, the type–species was not designated for the genus. This course of action makes the name unavailable under the provisions of the ICZN (1999: Article 67.4.1 which states: “A nominal genus-group taxon stablished after 1930 (or, in the case of an ichnotaxon, after 1999 [Art. 66.1]) must have its type species fixed in the original publication [Art. 13.3]”. As the nomenclature within the family Microparasellidae is well established and widely used, a proposition to the ICZN Commission is needed for maintaining the stability of the nomenclature and related authorities. The same situation exists for the genus *Microcharon* Karaman, 1934, now placed in the new family Lepidocharontidae. This state of affairs, along with the minimal standard of species descriptions, would require a detailed re-examination of the *Microparasellus* as a whole. Pending this procedure, authorities and dates of common use are hereby maintained.

The Microparasellidae can be regarded as monophyletic as here defined. While we do not enter a discussion into monophyly or otherwise of the Janiroidea, it is apparent, as shown by Wilson’s (1994) analysis, that the Microparasellidae lacks the characteristic operculate pleopods shown by the Janiroidea.

##### Distribution.

Species of the family are known from North Africa and eastern Europe only.
